# LIGHT deficiency attenuates acute kidney disease development in an in vivo experimental renal ischemia and reperfusion injury model

**DOI:** 10.1038/s41420-022-01188-x

**Published:** 2022-09-26

**Authors:** Quan-you Zheng, You Li, Shen-ju Liang, Xi-ming Chen, Ming Tang, Zheng-sheng Rao, Gui-qing Li, Jian-Li Feng, Yu Zhong, Jian Chen, Gui-lian Xu, Ke-qin Zhang

**Affiliations:** 1 Department of Urology, The 958th Hospital, The First Affiliated Hospital, Army Medical University, Chongqing, 400020 China; 2 Department of Immunology, Army Medical University, Chongqing, 400038 China; 3 Department of Nephrology, The First Affiliated Hospital, Army Medical University, Chongqing, 400038 China; 4 Department of ICU, The Third Affiliated Hospital, Army Medical University, Chongqing, 400042 China; 5 Department of Rheumatism and Immunology, The Third Affiliated Hospital, Army Medical University, Chongqing, 400042 China; 6 Urinary Nephropathy Center, The Second Affiliated Hospital, Chongqing Medical University, Chongqing, 400065 China

**Keywords:** Acute kidney injury, Renal fibrosis

## Abstract

Ischemia-reperfusion (I/R), a leading risk factor of acute kidney injury (AKI), is associated with high mortality and risk of progression to chronic kidney disease. However, the molecular mechanism of I/R-AKI remains not fully understood, which hinders its efficient clinical treatment. In this study, we observed that LIGHT deficiency remarkably attenuated I/R-AKI, as evidenced by rescued renal function, ameliorated tubular cell apoptosis, and alleviated inflammatory responses. Consistently, blocking LIGHT signaling with its soluble receptor fusion proteins (HVEM-IgG-Fc or LTβR-IgG-Fc) improved I/R renal dysfunction. RNA-sequencing and corresponding results indicated that LIGHT promoted oxidative stress and inflammation triggered by ischemic injury. Moreover, LIGHT signaling augmented ischemic stress-induced mitochondrial dysfunction characterized by an imbalance in mitochondrial fission and fusion, decreased mtDNA copies, impaired mitophagy, and increased mitochondrial membrane potential (ΔΨm). Mechanistically, LIGHT promoted mitochondrial fission by enhancing Drp1 phosphorylation (Ser616) and its translocation to the mitochondria. In conclusion, these results suggest that LIGHT-HVEM/LTβR signaling is critical for the I/R-AKI pathogenesis and it is further confirmed to be related to the increase in I/R-induced oxidative stress and mitochondria dysfunction, which may be the underlying mechanism of LIGHT signaling-mediated I/R-AKI.

## Introduction

Acute kidney injury (AKI), a common clinical syndrome, is characterized by an abrupt decline in renal function, with associated high mortality and morbidity [1, 2]. It can be caused by cardiac surgery, renal transplantation, sepsis, or nephrotoxic drugs. Furthermore, ischemia-reperfusion injury (I/R) is one of the leading risk factors [3]. Besides high short-term mortality, AKI is also an essential promoter of the progression of chronic kidney disease [4–7]. Despite ample clinical evidence for the improvement of renal function by renal replacement therapy or nutritional supplementation, until recently, no effective therapy has been established [8, 9]. Therefore, novel targets and potent therapeutic strategies for AKI are urgently needed.

The pathophysiology of I/R-induced AKI (I/R-AKI) is multifactorial and not fully understood, and several critical events, such as oxidative stress, inflammation, and tubular epithelial cell injury, have been proposed to mediate the I/R-AKI process [10–12]. Disruption of the cellular redox balance and excessive reactive oxygen species (ROS) production upon I/R-AKI injury triggers a series of events, including mitochondrial damage, energy depletion, inflammation, renal epithelial apoptosis, and necrosis [13–15]. Noteworthy, mitochondrial dysfunction has been defined as a convergence point among the pathological pathways of I/R-AKI [16, 17].

Several studies have indicated that mitochondria are dynamic organelles that constantly undergo a coordinated process of fission and fusion to maintain their morphology and normal function [18, 19]. In contrast to mitochondrial fusion, which is related to augmented ATP production, mitochondrial fission leads to detrimental oxidative phosphorylation and cellular injury [20, 21]. Dynamic related protein 1 (Drp1), an essential regulator of mitochondrial fission, promotes mitochondrial fission by translocating onto the mitochondrial outer membrane (MOM) and interacting with mitochondrial fission factor (MFF) and fission 1 protein (Fis1) under stress stimulation [22]. Furthermore, mitofusion 1/2 (Mfn1/2) and optic atrophy 1 (Opa1), fusion-related proteins, promote mitochondrial fusion by translocating to the MOM and mitochondrial inner membrane (MIM), respectively [23]. Dysregulated mitochondrial dynamics are closely linked to the pathogenesis of I/R-AKI [24, 25]. Moreover, an increasing number of studies have confirmed that mitochondrial fragment inhibition protects against various types of injury, including I/R-AKI [26, 27]. However, the molecular mechanisms that lead to mitochondrial fragmentation and dysfunction are unclear.

LIGHT (homologous to lymphotoxins) exhibits inducible expression and competes with HSV glycoprotein D for HVEM, a receptor expressed by T lymphocytes, also called TNFSF14 (tumor necrosis factor family member 14), or CD258, a novel member of the TNF superfamily, and plays a vital role in regulating both innate and adaptive immune responses by binding to its two receptors, lymphotoxin β receptor (LTβR) and HVEM [28, 29]. Growing evidence has confirmed the critical effect of LIGHT signaling in regulating inflammatory diseases, including pulmonary fibrosis, asthmatic airway remodeling, skin fibrosis, and rheumatoid arthritis [30–34]. Consistent with these results, our recent work supports the vital role of LIGHT in promoting renal fibrosis by enhancing sphk1 expression [35]. In contrast to the protective role of LIGHT in cisplatin-induced AKI by increasing mitochondrial apoptosis [36, 37], we found that LIGHT promotes sepsis-induced kidney injury through the TLR4-MyD88-NF-κB signaling pathway [36, 37]. However, it is unclear whether LIGHT signaling participates in regulating the mitochondrial fragmentation and function, and then affects the I/R-AKI pathology.

In this study, we aimed to uncover the precise effects and mechanisms of the LIGHT signaling in mediating I/R-AKI. Here, for the first time, we provide solid evidence that the LIGHT-HVEM/LTβR pathway triggers I/R-AKI, and it may be related to the increased oxidative stress and mitochondrial disorders triggered by I/R. Blocking the LIGHT signaling represents a promising strategy for the clinical treatment of I/R-AKI.

## Results

### LIGHT signaling promotes I/R-AKI

**Fig. 1:**
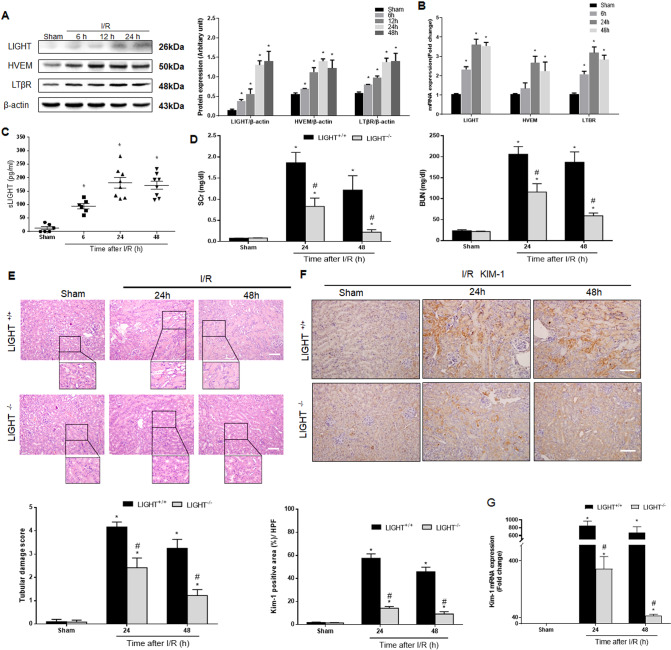
LIGHT-dependent pathway is required for the pathogenesis of ischemia-reperfusion-induced acute kidney injury. **A**–**C** LIGHT^+/+^ mice received 35 min of renal ischemia (clapping bilateral renal pedicles) followed by reperfusion for indicated time points. LIGHT and its receptor (HVEM and LTβR) expression were assessed by WB (**A**) and qPCR (**B**). **C** Plasma LIGHT concentration was tested by ELISA. (*n* = 6–8 for each group). **D**–**G** Wild-type (LIGHT^+/+^) and LIGHT knockout (LIGHT^−/−^) mice were conducted with renal ischemia followed by reperfusion for 24 or 48 h. **D** Measurements of serum Scr and BUN. **E** H&E staining and quantification of tubular damage score. Scale bar, 50 μm. **F** IHC staining and quantification for Kim-1 expression in kidney sections. Scale bar, 50 μm. **G** Kim-1 mRNA levels in renal tissue were tested. Values are presented as mean ± SEM. (*n* = 6–8 for each group). Differences between the two groups were assessed with Student’s *t* test. *p < 0.05 vs. sham group; ^**#**^*p* < 0.05 vs. LIGHT^+/+^ mice with I/R-AKI.

First, we tested the expression of LIGHT and its receptors (HVEM and LTβR) triggered by I/R-AKI. Followed by 35 min of bilateral renal pedicle clapping, LIGHT^+/+^ kidneys were subjected to reperfusion for several time points (6, 12, 24, or 48 h). The expression of LIGHT and its receptors rapidly increased and peaked 24 h after I/R surgery, according to western blotting (WB), quantitative reverse transcription-polymerase chain reaction (qRT-PCR), and immunohistochemistry (IHC) analyses (Fig. 1A, B, and Fig. S1). Similarly, serum LIGHT levels detected by ELISA were also remarkably upregulated after I/R surgery (Fig. 1C). Consistent with the murine I/R model results, LIGHT and expression of its receptors HVEM/LTβR in pathologically confirmed human acute epithelial injured renal tissues were significantly increased compared to healthy controls (Fig. S2). These results indicate that LIGHT pathways could play a critical role in the pathology of I/R-AKI.

Next, we used LIGHT knockout mice to explore the effect of LIGHT in I/R-AKI. Under normal conditions, comparable serum Cre and BUN levels were observed between LIGHT^+/+^ and LIGHT^−/−^ mice (Fig. 1D). Compared with the sham group, I/R-AKI induced significantly increased serum Cre and BUN levels and enhanced renal histological lesions, including brush border loss, tubular dilation, cast formation, tubular degeneration, and cell lysis (Fig. 1D, E). Based on the quantification, tubular damage scores also increased in I/R-AKI mice (Fig. 1E). LIGHT deficiency remarkably decreased Cre and BUN levels, attenuated renal histological lesions, and alleviated tubular damage scores (Fig. 1D, E). Consistently, kidney injury molecule 1 (KIM-1) expression was dramatically upregulated in I/R-stressed renal tissues, whereas LIGHT loss reversed these effects (Fig. 1F, G). Taken together, these findings reveal that the LIGHT-HVEM/LTβR pathway could contribute to the pathology of I/R-AKI.

### LIGHT pathway is critical for oxidative stress and inflammation in I/R-AKI

**Fig. 2:**
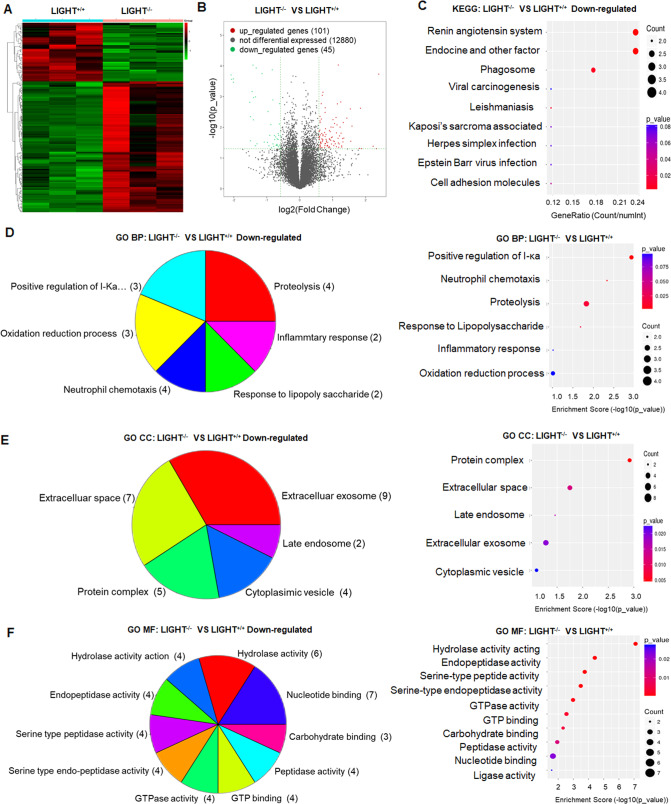
LIGHT deficiency affects RNA expression encoding the oxidation process, inflammation, and cell injury. Wild-type (LIGHT^+/+^) and LIGHT (LIGHT^−/−^) mice were conducted with renal ischemia followed by reperfusion for 24 h. Renal samples were collected and underwent transcriptome analysis. (*n* = 3 for each group). The heat map (**A**), volcano plot (**B**) graphs are shown. **C** KEGG analysis of downregulated genes in LIGHT^−/−^ mice, compared with that in LIGHT^+/+^ mice. **D**–**F** The Gene Ontology (GO) results of downregulated genes for LIGHT^+/+^ vs. LIGHT^−/−^. Differentially expressed genes (DEGs) were divided into three domains: biological process (BP), cellular components (CC), and molecular function (MF). Left panel: DEGs counts according to the classification strategy. Right panel: enrichment scores of DEGs.

To uncover the underlying mechanism by which the LIGHT pathway mediates I/R-AKI, kidneys were collected and tested with RNA-sequencing. Large-scale identification and functional categorization of differentially expressed genes (DEGs) were conducted. The heat map (Fig. 2A) and volcano plot graphs (Fig. 2B) identified a total of 146 DEGs. Then, the Gene Ontology (GO) of DEGs was assigned into three domains: Biological Process (BP), Cellular Component (CC), and Molecular Function (MF). As shown in Fig. 2D–F, LIGHT deficiency downregulated oxidative stress, compared to LIGHT^+/+^ mice. Additionally, LIGHT deficiency downregulated the expression of genes encoding positive regulation of I-κB, neutrophil chemotaxis, response to lipopolysaccharide, and inflammatory responses (Fig. 2D). Similar results were obtained from the KEGG analysis (Fig. 2C). These RNA-sequence results support the conclusion that the LIGHT pathway is critical for oxidative stress and inflammation in I/R-AKI pathogenesis.

### LIGHT deficiency attenuates I/R-induced oxidative stress

**Fig. 3:**
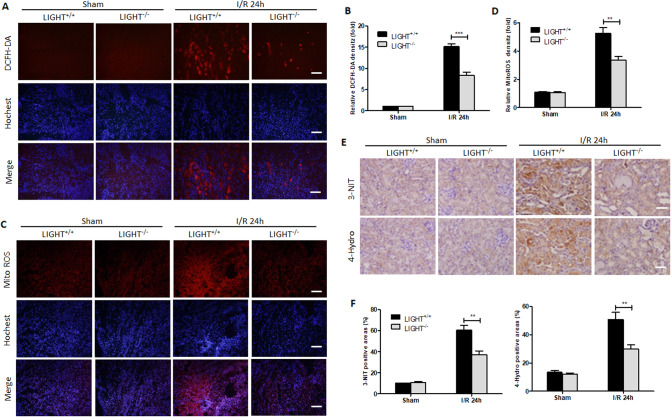
LIGHT deficiency attenuates ischemia-reperfusion-induced oxidative stress. Wild-type (LIGHT^+/+^) and LIGHT (LIGHT^−/−^) mice received renal ischemia followed by reperfusion for 24 h. **A** IF staining for DCFH-DA in frozen kidney sections. Scale bar, 50 μm **B** Quantification of DCFH-DA-positive areas per HPF. **C** IF staining for mitochondrial ROS. Scale bar, 50 μm. **D** Quantification of mtROS-positive areas per HPF. **E** 3-NIT and 4-hydro expression were tested by IHC. Scale bar, 50 μm. **F** Both 3-NIT and 4-hydro-positive areas were quantified. Values are presented as mean ± SEM. (*n* = 6–8 for each group). Differences between the two groups were assessed with Student’s *t* test. **p < 0.01; ***p < 0.001 vs. LIGHT^+/+^ mice with I/R-AKI.

The RNA-sequence results were further confirmed by I/R-induced excessive ROS and mtROS production in kidney tissues of LIGHT^+/+^ mice (Fig. 3A, C). Similarly, DCFH-DA and mtROS relative fluorescence intensity quantification in I/R mice showed remarkable increases compared to the sham groups (Fig. 3B, D). However, LIGHT deficiency significantly attenuated ROS and mtROS production (Fig. 3A–D). Additionally, IHC staining results showed that 3-NIT and 4-Hydro positive areas were increased in I/R surgery mice, whereas they were significantly decreased in LIGHT^−/−^ mice (Fig. 3E, F). Together, these findings indicate that the LIGHT pathway plays an essential effect in promoting I/R-induced oxidative stress.

### Deletion of LIGHT protects I/R-induced inflammation and renal tubular cell death

**Fig. 4:**
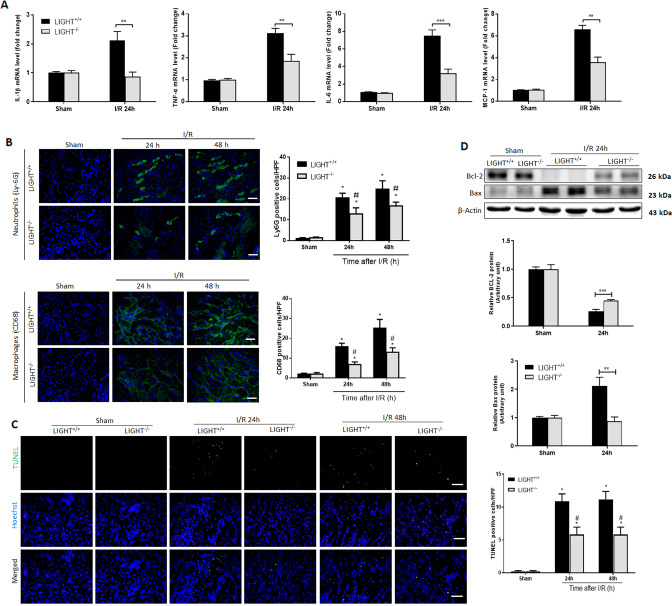
Deficiency of LIGHT reduces inflammation and tubular apoptosis after I/R injury. Wild-type (LIGHT^+/+^) and LIGHT (LIGHT^−/−^) mice received renal ischemia or sham surgery followed by reperfusion for indicated time points. **A** After 24 h reperfusion injury, kidney samples were assessed for indicated cytokine expression by qRT-PCR (*n* = 5–6). **B** Representative micrographs and quantification of immunofluorescence staining for neutrophils (Ly-6G) and macrophages (F4/80). The nucleus was co-stained with Hoechst 33258 (blue). (*n* = 5–6). Scale bar, 50 μm. **C** Western blotting for Bcl2 and Bax expression from kidney lysates after 24 h reperfusion injury, and a densito-metric analysis is shown in the underlying panel. **D** IF staining and quantification for TUNEL-positive apoptosis tubular cells. The nucleus was co-stained with DAPI (blue). Scale bar, 50 μm. Data are shown as mean ± SEM. (*n* = 6–8 for each group). Differences between the two groups were assessed with Student’s *t* test. *p < 0.05 vs. sham group; ^**#**^*p* < 0.05 vs. LIGHT^+/+^ mice with I/R-AKI.

An uncontrolled inflammation and renal tubular epithelial cell apoptosis promote the progression of I/R-AKI [1, 10]. Consistent with the published data and our RNA-sequence results, the degree of the renal inflammatory response, as evidenced by the expression of cytokines (IL-1β, TNF-α, IL-6, and MIP-1) and infiltration of Ly-6G neutrophils and F4/80 macrophages, was upregulated in renal tissues of I/R-AKI mice (Fig. 4A, B). Accordingly, LIGHT deficiency significantly mitigated inflammatory cytokine expression and neutrophil and macrophage infiltration (Fig. 4A, B). Moreover, TUNEL-positive apoptotic cells in renal tissues were remarkably increased and peaked at 24 h after the I/R challenge, as shown in Fig. 4C. However, I/R-induced TUNEL-positive apoptotic cells were reduced in LIGHT knockout mice (Fig. 4C). In accordance, I/R surgery significantly downregulated anti-apoptotic protein Bcl2 expression coupled with upregulated pro-apoptotic Bax expression compared with that of the sham group (Fig. 4D and Fig. S3). However, LIGHT loss partially counteracted these effects on Bcl2 and Bax expression (Fig. 4D). Overall, these results suggest that LIGHT promotes I/R-induced renal inflammatory responses and tubular cell apoptosis.

### LIGHT loss alleviates mitochondrial fragmentation while enhancing I/R injury-induced mitophagy

**Fig. 5:**
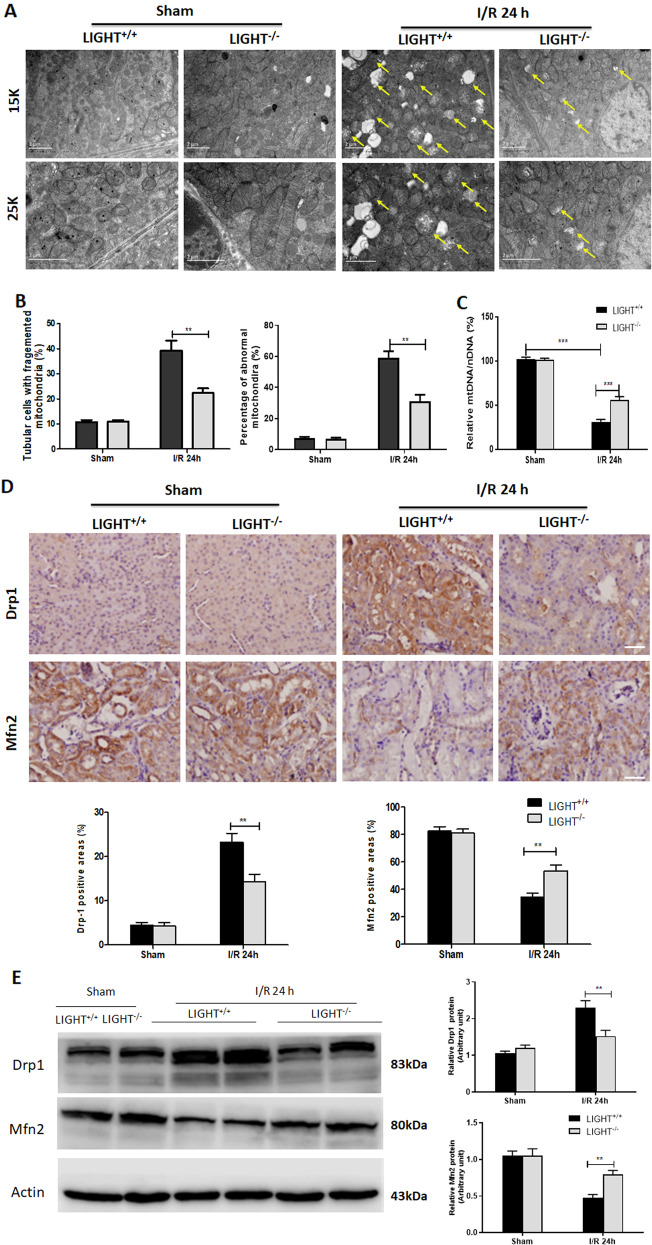
LIGHT loss decreases mitochondrial fragmentation. Wild-type (LIGHT^+/+^) and LIGHT (LIGHT^−/−^) mice received renal ischemia or sham surgery followed by reperfusion for 24 h. **A** Representative TEM images of mitochondrial morphology. Yellow arrows indicate the injured mitochondria in tubular cells. Scale bars: 2 μm. **B** Quantification of mitochondrial fragmentation and abnormal mitochondria detected by TEM. **C** Relative mitochondria DNA copy numbers (mtDNA/nDNA) assessed by qPCR. **D** IHC staining and quantification for the expression of Drp1 and Mfn2. Scale bar, 50 μm. **E** Immunoblot analysis of Drp1, Mfn2, and actin (loading control) in the renal tissue. Right pane: quantification of Drp1 and Mfn2 expression. Results are shown as mean ± SEM. (*n* = 6–8 for each group). Differences between the two groups were assessed with Student’s *t* test. *p < 0.05, **p < 0.01; ***p < 0.001 vs. LIGHT^+/+^ mice with I/R-AKI.

Mitochondrial damage, as evidenced by increased mtROS and epithelial tubular injury, was increased in the pathology of I/R-AKI and was closely related to the severity of renal inflammation and dysfunction [16, 17, 24]. To evaluate whether the LIGHT pathway affects mitochondrial fragmentation, mitochondrial ultrastructural changes were analyzed by electron microscopy. No difference in the mitochondria was found between LIGHT^+/+^ and LIGHT^−/−^ mice after sham I/R surgery (Fig. 5A, B). However, kidney tissue sections from I/R-AKI mice showed severe mitochondrial damage, including mitochondrial swelling and fragmentation (short/round), disruption of membrane integrity, vacuolization, and broken or absent cristae (Fig. 5A). Similar results were observed in the quantification of tubules with fragmented mitochondria and the percentage of abnormal mitochondria in I/R-injured kidneys (Fig. 5B). These defects were strongly ameliorated in LIGHT^−/−^ mice (Fig. 5A, B). Moreover, the significantly decreased mtDNA copy numbers in LIGHT^+/+^ mice under I/R stress were reversed in LIGHT^−/−^ mitochondria (Fig. 5C). IHC staining showed that the Drp1 was clearly enhanced, whereas the Mfn2 was decreased after I/R surgery, compared to that of sham groups (Fig. 5D). However, the upregulation of Drp1 was significantly reduced, but the downregulation of Mfn2 was restored in I/R kidneys of LIGHT^−/−^ mice compared with that of LIGHT^+/+^ mice (Fig. 5D). Similar results were obtained from western blots for Drp1 and Mfn2 expression (Fig. 5E). These results suggest that the LIGHT pathway promotes mitochondrial fragmentation under I/R stress.

Mitophagy protects against renal I/R injury by eliminating damaged mitochondria [38, 39]. To explore whether LIGHT affects mitophagy during I/R-AKI, we analyzed mitophagy-related proteins by WB. As shown in Fig. S4, mitophagy was activated upon ischemia/reperfusion stress, as evidenced by increased ULK1, and decreased TOMM20 compared with the normal condition. Accordingly, LIGHT knockout further enhanced mitophagy with significantly upregulated ULK1and downregulated TOMM20 expression than LIGHT^+/+^ mice (Fig. S3). Together, these results indicate that LIGHT deficiency ameliorates mitochondrial fragmentation coupled with enhanced mitophagy induced by I/R injury.

### Exogenous LIGHT promotes renal tubular cells oxidative stress and injury in vitro

**Fig. 6:**
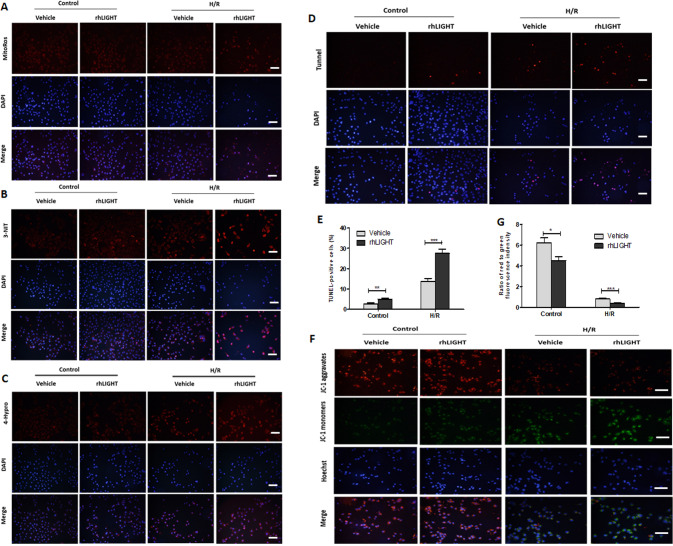
Exogenous LIGHT stimulation promotes oxidative stress and injury in vitro. HK2 cells were treated with a normal culture medium or recombinant human LIGHT and exposed to H/R conditions. For H/R condition, cells were subjected to 2 h OGR followed by 24 h reperfusion under normal oxygen conditions. Representative images of IF staining for mitochondria ROS (mtROS; **A**), 3-NIT (**B**), and 4-Hydro (**C**). Right panel: relative immunofluorescence density of mtROS, 3-NIT, and 4-Hydro. **D** Representative micrographs of TUNEL-positive apoptosis cells. **E** Quantification of TUNEL-positive cells is shown. **F** Membrane potential tested by JC-1 staining. Representative images of IF staining for mitochondria JC-1 aggravates (red) and monomers (green). **G** Ratio of red to green immunofluorescence density is shown. The nucleus was co-stained with DAPI (blue). Values are presented as mean ± SEM. Scale bar, 50 μm. Differences between the two groups were assessed with Student’s *t* test. *p < 0.05; **p < 0.01, ***p < 0.001.

We next focused on the effects of the exogenous LIGHT on tubular cell injury under H/R conditions using human proximal tubular cell line HK2 cells. Consistent with the published data [13], H/R-induced excessive oxidative stress in HK2 cells as reflected by upregulated mtROS, 3-NIT, and 4-Hydro expression compared with normal oxygen conditions, exogenous recombinant LIGHT stimulation further enhanced the oxidative stress (Fig. 6A–C). Moreover, increased cell apoptosis in the H/R condition was significantly promoted upon exogenous LIGHT stimulation (Fig. 6D, E and Fig. S5). Additionally, the mitochondrial membrane potential (ΔΨm) was impaired under H/R stress, and further enhanced upon exogenous LIGHT stimulation of HK2 cells (Fig. 6F, G). These data indicate that exogenous LIGHT stimulation promotes H/R-induced oxidative responses and HK2 cell injury.

### LIGHT stimulation enhances renal tubular cell mitochondrial fragments and Drp1 activation

**Fig. 7:**
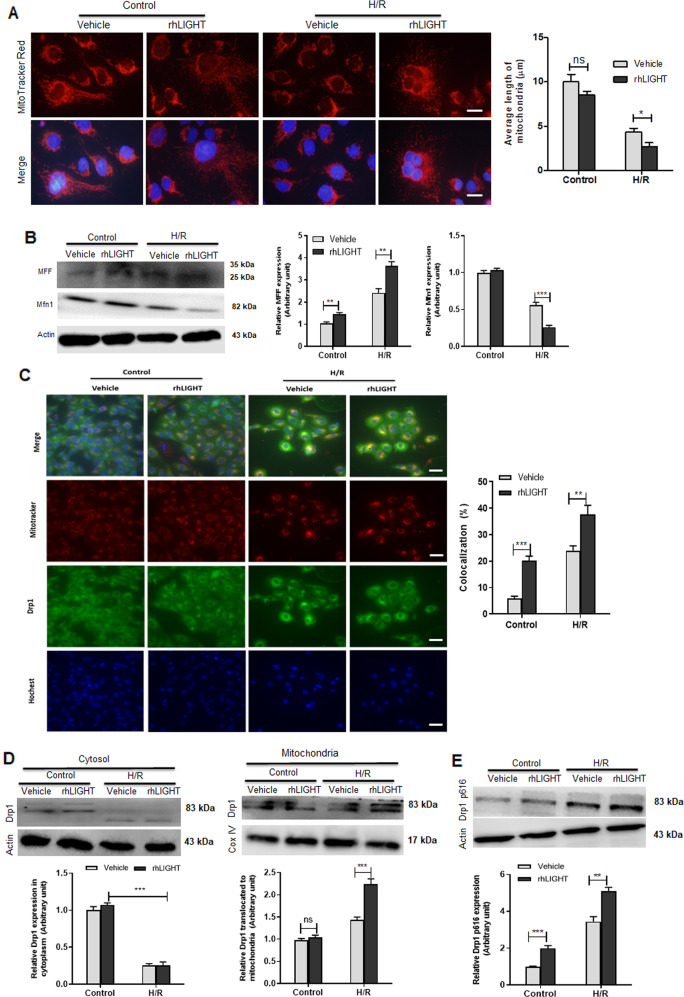
Exogenous LIGHT treatment enhances mitochondrial fragments and Drp1 activation. HK2 cells were treated with a normal culture medium or recombinant human LIGHT and exposed to H/R conditions. **A** Representative micrographs of immunofluorescence staining for mitochondrial fragments with Mito-Tracker Red. Right panel: average length of mitochondria was assessed to quantify the mitochondrial fragments. Scale bar, 10 μm. **B** Immunoblotting assay was used to examine the expression of mitochondrial fission (MFF) and fusion (Mfn1) proteins. Right panel: densitometric analysis is shown. **C** Representative micrographs of Drp1 in subcellular components following the H/R condition. Mitochondria were labeled with Mito-Tracker Red. Yellow staining indicates the binding of Drp1 to mitochondrial fragments. Right panel: quantification of Drp1 and Mito-Tracker Red colocalization are shown. The nucleus was co-stained with DAPI (blue). Scale bar, 20 μm. **D** Western blotting for Drp1 translocation from cytoplasm to mitochondria. Densitometric measurements of Drp1 expression in cytoplasm or mitochondria are shown underlying the pane. **E** Western blotting for Drp1 p616 phosphorylation. Densitometric measurements of Drp1 p616 phosphorylation are shown underlying the panel. Values are presented as mean ± SEM. Differences between the two groups were assessed with Student’s *t* test. ns, no significance, *p < 0.05; **p < 0.01, ***p < 0.001.

Mitochondrial fragmentation promotes I/R-induced tubular cell injury by increasing mtROS pro-apoptotic protein expression and mtDNA injury [25, 40]. To confirm the detrimental effect of LIGHT signaling on renal tubular cell mitochondrial debris in vivo, we tested the effects of exogenous LIGHT on mitochondrial fragmentation in HK2 cells under H/R stress. H/R stimulation produced smaller, rounder, and shorter fragmented mitochondria than those in the control group, and exogenous LIGHT exposure significantly increased mitochondrial fragmentation induced by H/R stress (Fig. 7A). Similar results were detected by western blot analysis (Fig. 7B). Additionally, IF staining for MFF and Mfn1 confirmed that LIGHT enhanced mitochondrial fragmentation (Fig. S6). Moreover, IF staining for Drp1 revealed that exogenous LIGHT treatment augmented H/R-induced mitochondrial debris compared to the controls (Fig. 7C).

Drp1 translocation, from the cytoplasm to the mitochondria, is an essential step in mitochondrial fission [16, 24]. The intracellular localization of Drp1 was assessed by WB. LIGHT stimulation significantly increased Drp1 translocation (Fig. 7D). To investigate the effects of LIGHT signaling on Drp1 activation under H/R stress, we tested the phosphorylation levels of Drp1 at Ser616 by WB. Consistent with the promoted translocation effects, LIGHT stimulation further upregulated H/R-induced the phosphorylation of Drp1 at Ser616 even under normal oxygen conditions (Fig. 7E). Taken together, these data suggest that LIGHT signaling could enhance renal tubular cell mitochondrial fragments by stimulating Drp1 activation at Ser616 and its translocation into mitochondria.

### Recombinant LIGHT decreases H/R-induced renal tubular cells mitophagy

Mitophagy attenuates renal I/R injury by eliminating damaged mitochondria [38, 39]. To determine the effects of LIGHT signaling on H/R-induced mitophagy in vitro, we analyzed HK2 cell mitophagy by IF staining and WB. It was shown that H/R stimulation enhanced cellular mitophagy, but exogenous LIGHT significantly reversed this effect (Fig. S7A). Contrast to comparable expression of BNIP3, WB results showed that enhanced mitophagy upon H/R stress was attenuated by LIGHT treatment (Fig. S7B). These results indicate that LIGHT signaling impairs H/R-induced mitophagy in vitro.

### Both LTβR and HVEM receptors are involved in I/R-induced AKI

**Fig. 8:**
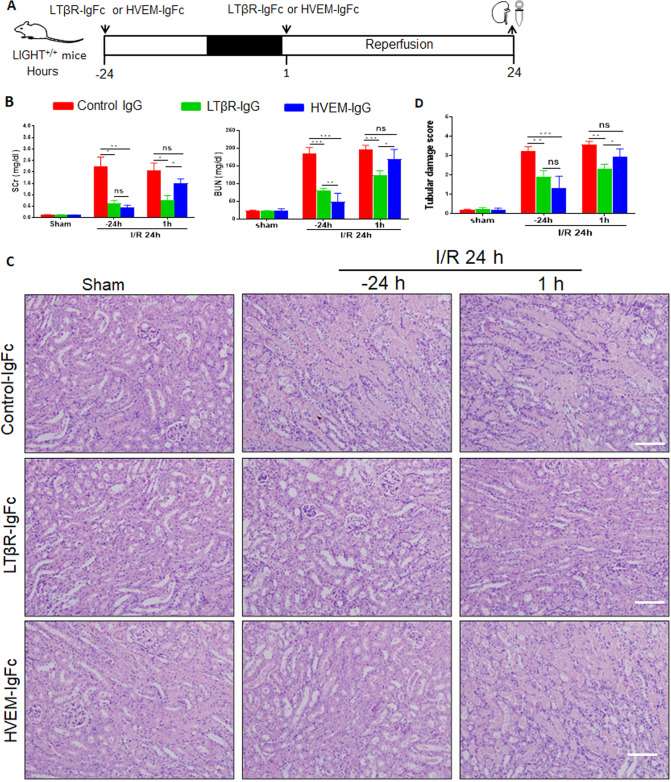
Blocking LIGHT signaling with soluble LTβR-IgG-Fc or HVEM-IgG-Fc fusion proteins protects against renal ischemia/reperfusion injury in mice. LIGHT^+/+^ mice pretreated with control IgG-Fc, soluble HVEM-IgG-Fc, or LTβR-IgG-Fc, followed by 35 min of renal ischemia and 24 h reperfusion. **A** A schematic graph for the procedures of I/R-AKI. **B** Measurement of serum Scr and BUN levels. **C** H&E staining for mice kidney sections. Scale bar, 50 μm. **D** Quantification of pathological scores of tubular damages. Values are presented as mean ± SEM. Differences between the two groups were assessed with Student’s *t* test. ns, no significance, *p < 0.05; **p < 0.01, ***p < 0.001.

To explore which receptor contributes to LIGHT signaling in the I/R-AKI process, we blocked LIGHT signaling with soluble LTβR-Ig-Fc or HVEM-Ig-Fc fusion proteins, as shown in a schematic description in Fig. 8A. Pretreatment for 24 h with LTβR-Ig-Fc or HVEM-Ig-Fc fusion proteins significantly alleviated I/R-induced renal function, with decreased serum Cre and BUN levels than control mice (Fig. 8B). Consistently, histological analyses of renal tissues showed much less severe epithelial tubular injury from fusion protein-treated mice (Fig. 8C, D). Moreover, despite the lack of effect on the renal injury of post-treatment 1 h after I/R surgery with HVEM-Ig-Fc fusion protein, LTβR blocking remarkably attenuated renal injury with significantly decreased serum Cre and BUN, and alleviated histological damages compared to that of the controls (Fig. 8B–D). Overall, these data showed that LIGHT mediates I/R-induced AKI through interaction with both LTβR and HVEM.

### LIGHT signaling loss alleviates I/R-induced renal fibrosis

**Fig. 9:**
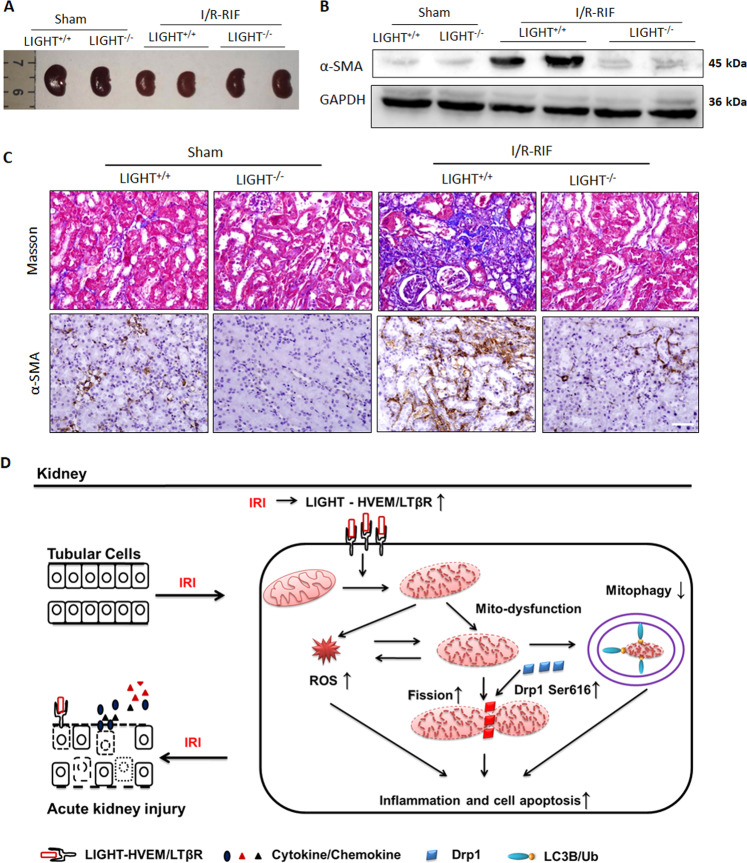
LIGHT deficiency attenuates I/R-induced renal interstitial fibrosis. LIGHT^+/+^ and LIGHT^−/−^ mice received 40 min of renal ischemia (clapping left renal pedicles) and the kidney was collected on day 28. **A** Gross-morphological changes of kidneys. **B** Immunoblot analysis of α-SMA in the renal tissue. **C** Masson and IHC staining (α-SMA) for renal fibrosis. Scale bar, 50 μm. **D** Schematic description of how the LIGHT-HVEM/ LTβR pathway mediates I/R-AKI by promoting oxidative stress and mitochondrial dysfunction.

Considering that AKI is also an essential promoter of the progression to chronic kidney disease [4–7], lastly, we investigated the effect of LIGHT signaling on long-term outcomes of I/R-AKI. The I/R surgery induced significant renal fibrosis at day 28, as evidenced by increased renal atrophy, upregulated pro-fibrotic protein α-SMA expression (WB and IHC staining), and augmented collagen deposition by staining with Masson’s trichrome (Fig. 9A–C). However, LIGHT deficiency remarkably alleviated renal atrophy, α-SMA expression, and Masson staining (Fig. 9A–C).

## Discussion

Numerous data has confirmed the essential role of LIGHT signaling in mediating several inflammatory diseases, including pulmonary fibrosis, inflammatory bowel diseases, skin fibrosis, and rheumatoid arthritis [31–34, 41]. However, the effects and underlying mechanisms of LIGHT signaling in the pathology of I/R-AKI remain not fully understood. In this study, using bilateral pedicle clamp-induced acute renal injury, LIGHT knockout or blocking the LIGHT signal with its soluble receptor fusion proteins HVEM-IgG-Fc or LTβR-IgG-Fc significantly ameliorated I/R-AKI. This is the first study to show that I/R-AKI, including renal function, tubular cell apoptosis, and inflammatory responses, was alleviated in LIGHT-deficient mice or by the LIGHT signal blockage. Accordingly, we showed that LIGHT loss attenuates I/R-induced renal fibrosis. RNA-sequence data indicated that the LIGHT signaling enhanced oxidative stress induced by ischemic injury. Moreover, LIGHT signaling augments mitochondrial dysfunction by disturbing the balance between mitochondrial fission and fusion, impairing mitophagy, promoting ΔΨm, and enhancing mtDNA injury-induced by I/R stress. Mechanistically, LIGHT could mediate mitochondrial fission by enhancing Drp1 phosphorylation and translocation to the mitochondria. Our study indicated that LIGHT-HVEM/LTβR could mediate renal ischemia-reperfusion-induced acute injury by enhancing oxidative stress and mitochondrial dysfunction, as shown in the schematic in Fig. 9D.

LIGHT is an essential co-stimulatory molecule that mediates innate and adaptive immune responses and contributes to some inflammatory disorders [32, 33, 41]. Renal tissues constitutively synthesize LIGHT, and LIGHT and its receptor expression are upregulated during inflammatory kidney diseases relative to the stable stage, indicating that LIGHT signaling regulates renal homeostasis [28, 35]. Our recent work supports that LIGHT mediates unilateral ureteral occlusion-induced renal interstitial fibrosis by increasing the expression of the pro-fibrotic protein sphk1 [35]. Consistently, LIGHT signaling promotes I/R-induced renal fibrosis in this study. However, the mechanism remains elusive, which needs further investigation. Additional studies revealed that LIGHT promotes sepsis-associated kidney injury by enhancing the TLR4-MyD88-NF-κB signaling pathway [37]. Consistently, this study indicates that LIGHT signaling mediates renal IRI by enhancing oxidative stress and promoting mitochondrial dysfunction. Mechanistic studies indicate that LIGHT signaling upregulates Drp1 phosphorylation and translocation into the mitochondria, contributing to mitochondrial fission and fragmentation. However, in addition to multiple studies supporting the detrimental role of the LIGHT pathway in several inflammatory diseases as described above, the protective effect of this molecule in inflammatory bowel disease and beige fat biogenesis by inhibiting inflammatory cytokine expression [31, 33, 34, 41, 42] and in cisplatin-induced AKI by ameliorating mitochondrial apoptosis have been reported [36]. The differences in diverse disease pathophysiology, genetic backgrounds in model animals, and certain inflammatory responses might contribute to these disparate findings. Further research is needed to resolve these discrepancies.

Ischemia-reperfusion-induced excessive ROS generation and the disruption of the cellular redox balance contribute to several events, including energy depletion, inflammation, renal tubular cell injury, and mitochondrial dysfunction, which ultimately mediate acute renal injury or impaired recovery [10, 16]. Consistently, the RNA-sequence analysis suggested that the attenuated oxidative stress and increased oxidative reduction process might contribute to reversed renal function and tubular cell injury in LIGHT-deficient mice. These results were confirmed by downregulated oxidative stress marker expression, including ROS, mtROS, 3-NIT, and 4-Hydro in LIGHT-deficient mice. Consistent with these in vivo data, recombinant LIGHT stimulation further enhanced H/R-induced oxidative stress in vitro, as evidenced by upregulation of mtROS, 3-NIT, and 4-Hydro expression (Fig. 6A–F). Moreover, a recent study revealed that inhibition of mtROS production significantly alleviated ischemia AKI by rescuing mitochondrial function and attenuating inflammation [13, 43]. Together, these data indicate that LIGHT signaling could mediate I/R-AKI by enhancing oxidative stress.

LIGHT signaling regulates inflammatory responses and cell death [37, 44]. Consistently, the RNA-sequence data in the present study indicated that LIGHT deficiency remarkably decreased the expression of some genes encoding positive regulation of I-κB, neutrophil chemotaxis, response to LPS, and inflammatory responses (Fig. 2A). These RNA-sequence results were further confirmed by downregulation of cytokine expression (IL-1β, TNF-α, IL-6, and MCP-1), suppressed neutrophil and macrophage infiltration, decreased tubular cellular apoptosis, and disturbed expression of pro- and anti-apoptotic proteins in ischemic injured LIGHT loss kidneys. Additionally, exogenous recombinant LIGHT stimulation further increased H/R-induced HK2 cell apoptosis, as shown in Fig. 6D. Overall, these results suggest that LIGHT signaling promotes ischemia-reperfusion-induced cell death and inflammation. However, the downstream signaling pathway involved needs to be investigated in the future.

Increasing evidence suggests that mitochondrial damage and dysfunction mediate the pathogenesis of several acute kidney injuries, including I/R-AKI [16, 17]. However, the underlying mechanisms that lead to mitochondrial dysfunction remain unclear. Our previous study showed that LIGHT signaling regulates pancreatic beta cells and renal tubular cell death by promoting mitochondrial apoptosis [37, 45]. Therefore, we hypothesized that the LIGHT pathway could play a vital role in the progression of I/R-AKI by regulating mitochondrial function. Next, the mitochondrial ultrastructure and dynamic-associated proteins were examined. It was shown that LIGHT deficiency preserved mitochondrial morphology, rescued mtDNA copy numbers, attenuated mitochondrial fission, and preserved mitochondrial fusion in the I/R-AKI model (Fig. 5). Moreover, we found that LIGHT loss remarkably increased mitophagy induced by I/R (Fig. S3). Consistent with this, this study further confirmed the detrimental effect of LIGHT signaling in promoting mitochondrial dynamics and inhibiting mitophagy in HK2 cells under H/R conditions in vitro (Fig. 7, Figs. S5, and Fig. S6). Additionally, H/R stress reduced ΔΨm, further impaired upon recombinant LIGHT stimulation in HK2 cells (Fig. 6F). Collectively, these data suggest that LIGHT signaling mediates ischemic acute renal injury by enhancing mitochondrial dysfunction and inhibiting mitophagy.

Mitochondrial fission plays a vital role in mediating ischemia-induced acute renal disease [24]. Drp1, an essential initiator of mitochondrial fission, is regulated by several post-transcriptional modifications, including phosphorylation [46]. The effects of LIGHT signaling on Drp1 activation and translocation were investigated in this study. This is the first study to show that LIGHT stimulation promotes Drp1 translocation from the cytoplasm into the mitochondria in HK2 cells under H/R conditions. Consistently, our results indicate that LIGHT treatment enhances Drp1 activity by upregulating phosphorylation at Ser616, even under normal conditions. In addition to phosphorylation, Drp1 activity is regulated by SUMOylation and acetylation [47, 48]. Whether LIGHT mediates Drp1 SUMOylation or acetylation needs to be further investigated.

In conclusion, our study supports the pivotal effect of the LIGHT-HVEM/LTβR signaling in mediating I/R-AKI and I/R-induced renal fibrosis. Mechanistic studies have shown that LIGHT signaling promotes I/R-triggered oxidative stress and mitochondrial dysfunction. LIGHT could enhance mitochondrial fission by enhancing Drp1 phosphorylation at Ser616. Finally, blocking the LIGHT signaling with HVEM-IgG-Fc and LTβR-IgG-Fc rescued I/R-induced renal injury. Our results showed that blocking LIGHT-HVEM/LTβR pathway alleviates I/R-induced kidney injury, which further supports the potential clinical efficacy of LIGHT inhibitors for I/R-AKI in humans.

## Materials and methods

### Animal experiments

LIGHT-deficient (LIGHT^−/−^, C57BL/6 J background) mice were obtained from Prof. Pfeffer (Institute of Medical Microbiology and Hospital Hygiene, University of Duesseldorf, Germany). Wild-type (WT) (LIGHT^+/+^, C57BL/6 J background) mice were purchased from the Animal Institute of the Academy of Medical Science (Beijing, China). Mice were housed in a specific-pathogen-free (SPF) facility with free access to food and water. Male mice aged 8–12 weeks were used. Animal experiments were conducted according to the guidelines of the Institutional Animal Care and Use Committee of the Army Medical University (Third Military Medical University).

The I/R-AKI experiment was performed according to the published protocols [49]. Briefly, mice were anesthetized with 1% pentobarbital (i. p., 50 mg/kg), and a midline incision exposed the bilateral renal pedicles. The renal pedicles were bilaterally clipped with microvascular clamps for 35 min, and then removed the clamps for reperfusion at the indicated time points (6, 12, 24, or 48 h). Mice that underwent the same surgery without renal pedicle clipping were regarded as the sham controls. For blocking experiments, LIGHT^+/+^ mice were randomly divided into three groups (*n* = 6): control human IgG-Fc (Sino Biotechnology Inc.) group; HVEM-IgG (the murine extramembrane HVEM fused with human IgG-Fc fragment, Sino Biotechnology, lnc.) group; and LTβR-IgG (the murine extramembrane LTβR fused with human IgG-Fc fragment, Zoonbio Biotechnology, Co.) group. Mice were intraperitoneally injected with control human IgG-Fc (100 μg), HVEM-IgG (100 μg), or LTβR-IgG (100 μg) 24 h before or 1 h after renal pedicle clamping. After 24 h of reperfusion, the mice were sacrificed, and kidneys were collected. For I/R-induced renal fibrosis, the left renal pedicle was clipped with microvascular clamps for 40 min and the kidney samples were collected at day 28. All experiments were blinded to I/R induction and experimental results.

### Assessment of renal function

Serum creatinine (Scr) and blood urea nitrogen (BUN) levels were measured utilizing a biochemical auto-analyzer system (Olympus AU5400, Japan).

### ELISA and histopathological analysis

The levels of serum LIGHT in the sham and I/R groups were measured with the Mouse TNFSF14 ELISA Kit (SEA827Mu, Cloud-Clone Corp, Wuhan, China). For histopathological assessments, kidneys were fixed 4% paraformaldehyde and immersed into paraffin. Prepared renal sections (4 μm) were stained with hematoxylin and eosin (H&E) or Masson’s trichrome and analyzed using an Olympus microscope (Tokyo, Japan). Damaged renal tubules were defined as cast formation, tubular dilation and disruption, cell lysis, or brush border loss. Tubular damage scores were assessed by the percentage of injured tubules: 0, no damage; 1, <25%; 2, 25–50%; 3, 50–75%; and 4, >75%. At least six viewing fields were selected and examined for each section, and the semi-quantitative analysis was performed in a blinded fashion by two experienced individuals.

### QRT-PCR analysis

Total RNA was extracted from the indicated renal tissues utilizing TRIzol reagent (Biomed, Beijing, China). PrimeScript^TM^ RT Reagent Kit (Takara, Shiga, Japan) was used to synthesize cDNA. Real-time PCR was performed using SYBR^®^ Premix Ex Taq^TM^ Reagent Kit (Takara, Shiga, Japan) on MxPro3000P (Agilent StrataGene, USA). The 25 μL qPCR reaction mix contained 2 μL cDNA and 0.5 μmol primer pairs for the target genes or GAPDH (Table 1). Amplification was conducted in triplicate. The samples were normalized to GAPDH levels. Gene expression was quantified using the ∆∆Ct method [50].

**Table 1:** mRNA sequences.

Gene	Primer sequence
LIGHT forward	5′-TGGCTCCTGTAAGATGTGCTG-3′
LIGHT reverse	5′-GTTTCTCCTGAGACTGCATCAA-3′
LTβR forward	5′-TGCATACCGCAAAGACAAACTC-3′
LTβR reverse	5′-TGGTGCCCCCTTATCGCATA-3′
GAPDH forward	5′-ACCACAGTCCATGCCATCAC-3′
GAPDH reverse	5′-TCCACCACCCTGTTGCTGTA-3′
HVEM forward	5′- ACTCGTCTCCCACAAGGAACT-3′
HVEM reverse	5′-CAGGCCCCTACAGACAACAC-3′
IL-6 forward	5′-GCCCTTCAGGAACAGCTATGA-3′
IL-6 reverse	5′-TGTCAACAACATCAGTCCCAAGA-3′
KIM-1 forward	5′-TTCTCTGTACCATGACACTCTGC-3′
KIM-1 reverse	5′-ACAAGCAGAAGATGGGCATTG-3′
MCP-1 forward	5′-TTAAAAACCTGGATCGGAACCAA-3′
MCP-1 reverse	5′-GCATTAGCTTCAGATTTACGGGT-3′
TNF-α forward	5′-TCTTCTCATTCCTGCTTGTGG-3′
TNF-α reverse	5′-GGTCTGGGCCATAGAACTGA-3′
ND-1 forward	5′-CTAATCGCCATAGCCTTCCTAA-3′
ND-1 reverse	5′-GTTGTTAAAGGGCGTATTGGTT-3′
LPL forward	5′-CCTGATGACGCTGATTTTGTAG-3′
LPL reverse	5′-CAATGAAGAGATGAATGGAGCG-3′

### RNA sequence

RNA sequencing was performed with an Illumina Hiseq 4000 sequencer, as described in the provided instructions. Briefly, total RNA was extracted from kidneys with TRIzol and then subjected to quality testing and quantification using a Nanodrop ND-1000 (Nanodrop, USA). After enrichment with oligo (dT) magnetic beads, mRNA was used to synthesize cDNA with random primers and dUTP-based primers. The synthesized cDNA was terminally repaired with A, Illumina connectors were added, and DNA libraries were built using PCR amplification. Following quality testing using the Agilent 2100, RNA sequencing was performed using the Illumina Hiseq 4000. Solexa Pipeline Version 1.8 was used for image visualization and base recognition, FastQC for quality evaluation, and Hisat2 software for comparison with the reference genome. Stringtie and Ballgown software were used to estimate the transcriptional abundance with reference to the official annotated database and to calculate the FPKM values at the gene and transcriptional levels, respectively. Differences in gene expression and transcriptional levels were calculated, and screening of DEGs among samples or groups was determined. The gene expression level, PCA, correlation, cluster analysis, GO (http://www.geneontology.org), and pathway functional significance enrichment analyses were conducted for the DEGs. The ontology was divided into three domains: BP, CC, and MF. Significant DEGs were defined with a Benjamini and Hochberg corrected *p* value with a cutoff at 0.05 and fold-change of at least 1.5. The lower the *p* value, the more significant the GO term (*p* value ≤ 0.05 is recommended).

### Human kidney tissues

Human kidney tissues were obtained from patients (*n* = 10) only after histological confirmation of acute tubular injury. Normal control renal tissues were collected from patients diagnosed with renal cell carcinoma who received radical nephrectomy, and normal peritumoral tissue was collected (*n* = 5). Human sample utilization was approved by the Institutional Ethical Board of the Army Medical University, and all subjects had signed written informed consent.

### Immunohistochemical analysis

Paraffin kidney sections from mice or humans (4 μm) were deparaffinized and rehydrated, and antigen retrieval was conduction utilizing 10 mM sodium citrate or EDTA for 15 min within a microwave oven. After blocking with 3% H_2_O_2_/PBS and 5% bovine serum albumin (BSA)/PBS, the following primary antibodies were incubated overnight at 4 °C: rabbit mouse anti-human/mouse LIGHT, LTβR, KIM-1, 3-NIT, 4-Hydro, Drp1, Mfn2, Bcl2, α-SMA (1:200, Abcam, Cambridge, UK), and rabbit anti-mouse/human HVEM (1:200, Santa Cruz, USA). Sections were then incubated with horseradish peroxidase-labeled goat anti-rabbit/mouse secondary antibodies (1:800, Beyotime, Shanghai, China) for 1 h at 25 °C. Following washing three times with PBS, 3,3’-diaminobenzidine chromogen solution (DAB, Beyotime, Shanghai, China) was used for visualization, and the sections were counterstained with hematoxylin and analyzed with light microscopy (Olympus, Tokyo, Japan). At least six viewing fields were randomly selected to quantify the number of positive areas (0.04 mm^2^).

### Immunofluorescence staining

Kidney cryosections (8 μm) were immersed into cold acetone for 15 min and then blocked with 5% BSA for 1 h at 25 °C. After incubated with primary antibodies against Ly-6G or F4/80 (1:100, Abcam, Cambridge, UK) overnight at 4 °C, sections were incubated with Dylight488-conjugated secondary antibody (1:200, Biolegend, San Diego, CA, USA), and then the nuclei were co-stained with Hoechst 33258 (5 μg/mL, Beyotime, Shanghai, China). The cryosections were tested using fluorescence microscopy (Olympus, Tokyo, Japan). For ICC experiments, the indicated coverslips were fixed with 4% paraformaldehyde, permeabilized with 0.1% TritonX-100, and blocked with 5% BSA. The slips were incubated with primary antibodies against 3-NIT, 4-Hydro, Drp1, Mfn1, or MFF (1:100, Abcam, Cambridge, UK) overnight at 4 °C. In some experiments, mitochondria were labeled with Mito-Tracker Red CMXRos before fixation (Beyotime, Shanghai, China) according to the provided protocols. The next day, slips were stained with Cy3/ Dylight488-conjugated secondary antibody (1:200, Biolegend, San Diego, CA, USA), and then nuclei were co-stained with Hoechst 33258 (5 μg/mL, Beyotime, Shanghai, China). Quantification of positively stained cells was performed as described previously.

### Transmission electron microscopy

For transmission electron microscopy (TEM) analysis, renal cortices (1 mm^3^) were rapidly fixed with 2.5% glutaraldehyde for 1 h at 25 °C and stored at 4 °C overnight. Renal cortices were post-fixed in 2% osmium tetroxide, dehydrated with graded ethanol, immersed into hard resin, and then prepared ultra-thin sections utilizing a Leica ultra-microtome (Leica, Germany). These sections were stained with uranyl acetate and lead citrate, and observed using a TEM instrument (Hitachi, HT7700, Japan). Mitochondria with a length less than 1 μm were considered fragmented.

### mtDNA copy number testing

Total renal cortex DNA was extracted with a DNA Extraction Kit (Invitrogen, USA). Both mtDNA (evaluated by the mitochondrial ND-1 gene) and nDNA (evaluated by the LPL gene) were tested by quantitative real-time PCR using SYBR^®^ Premix Ex Taq^TM^ Reagent Kit (Takara, Shiga, Japan) on MxPro3000P (Agilent StrataGene, USA). The primer pairs used are listed in Table 1.

### Cell culture and stimulation

Human proximal tubular cells (HK2) were purchased from the American Type Culture Collection (ATCC, Manassas, VA, USA), and cultured in complete DMEM/F12 medium (Hyclone, USA) supplemented with100 μg/mL streptomycin, 100 U/mL penicillin, and 10% FBS (Gibco) in a humidified incubator (5% CO_2_ at 37 °C). For the H/R model, cells were cultured in serum-free F12 medium for 24 h, replaced with serum- and glucose-free DMEM medium (Solarbio, Beijing, China), and transferred into a hypoxia (1% O_2_, 5% CO_2_, and 94% N_2_) incubator chamber (Stem cell, UK) for additional 2 h. Cells were then transferred into normoxic conditions (21% O_2_, 5% CO_2_, and 74% N_2_) with a fresh complete F12 culture medium supplemented with recombinant human LIGHT (5 μg/mL, PeproTech, USA) for 24 h to allow reoxygenation and stimulation. Cells without H/R treatment were used as controls.

### Western blot analysis

Renal tissue and HK2 cells were homogenized and lysed with T-PER solution containing protease and a phosphatase inhibitor cocktail (Roche, Basel, Switzerland) for 30 min on ice. Mitochondria were isolated from HK2 cells by differential centrifugation utilizing a commercial Mitochondria Isolation Kit (Beyotime, Shanghai, China). Following quantification with a BCA Kit (Beyotime, Shanghai, China), the indicated protein samples (30 μg) were fractionated by 10–12% SDS-PAGE gels and transferred to polyvinylidene difluoride membranes (PVDFs, Millipore, Billerica, MA, USA). After blocking with 5% non-fat milk for 60 min, the PVDFs were incubated with primary antibodies against rabbit/mouse anti-human/mouse LIGHT, LTβR, Bcl2, Drp1, Bcl2, Parkin, TOMM20, ULK1, and α-SMA (1:500, Abcam, Cambridge, UK), rabbit anti-mouse/human MFF, Mfn1, Mfn2, phosphor-Drp1 Ser616, BNIP3 (1:500, CST, Danvers, MA, USA), rabbit anti-mouse/human HVEM (1:800, Santa Cruz, USA), or rabbit anti-mouse Bak, β-actin (1:800; Beyotime, Shanghai, China) overnight at 4 °C. The next day, the blots were incubated with horseradish peroxidase-labeled secondary antibodies (1:1000, Beyotime, Shanghai, China). The blots were detected utilizing an Enhanced Chemiluminescence Kit (Solarbio, Beijing, China) and quantified with ImageJ software (NIH, Bethesda, MD, USA).

### TUNEL staining and annexin-FITC apoptosis detection

Deparaffinized and rehydrated kidney sections (4 µm) were stained with the Cell Death Detection Kit (Roche, Basel, Switzerland). For indicated HK2 coverslips, cells were stained with the Cell Meter™ Apoptosis Assay Kit (ATT Bioquest^®^, Sunnyvale, CA, USA). The coverslips were then incubated with Hoechst 33258 and analyzed using a confocal laser scanning microscope (Leica Microsystems, Wetzlar, Germany). TUNEL-stained cells were quantified with ImageJ software. HK2 cells were also collected after treatment, and then the percentage of apoptotic cells utilizing an Annexin V-FITC/PI Apoptosis Detection Kit (Biolegend, San Diego, CA, USA) was determined.

### Assessment of ROS

For ROS production analysis, kidney cryosections (8 μm) were incubated with a DCFH-DA fluorescent probe from the ROS Assess Kit (Beyotime, Shanghai, China). The levels of mtROS production in the kidney or HK2 cells were measured using MitoSOX Red (5 μM, Invitrogen) staining in accordance with the provided protocols. Briefly, kidney cryosections (8 μm) or indicated HK2 cells were incubated with MitoSOX Red in darkness at 37 °C for 15 min, co-stained with Hoechst 33258 (5 µg/mL) for 2 min, and then examined using a confocal laser scanning microscope (Leica Microsystems, Wetzlar, Germany). The positive cells were quantified using ImageJ software.

### Mitochondria membrane potential analysis

For ΔΨm analysis, HK2 coverslips were incubated with the Enhanced Mitochondria Membrane Potential Assay Kit with JC-1 probe (Beyotime, Shanghai, China). In brief, HK2 coverslips were stained with pre-warmed PBS containing JC-1 (1:1000) in darkness at 37 °C for 30 min. Following co-staining with Hoechst 33258 (5 µg/mL), the coverslips were analyzed with a confocal laser scanning microscope (Leica Microsystems, Wetzlar, Germany).

### Statisal analysis

Results are shown as the mean ± standard error of the mean. Statistical analyses were performed with GraphPad Prism 6.0 software (GraphPad Software, Inc., La Jolla, CA). Differences between the two groups were assessed with Student’s *t* test, and statistical significance was set at *p* < 0.05.

## Supplementary information

Supplementary material

Original Data File

## Data Availability

All data generated or analyzed during this study are available from the first or corresponding author upon reasonable request.
