# Supplementary material for: LIGHT deficiency attenuates acute kidney disease development in an in vivo experimental renal ischemia and reperfusion injury model
Source: Cell Death Discov. 2022 Sep 26;8:399. doi: 10.1038/s41420-022-01188-x (PMC9512920; doi:10.1038/s41420-022-01188-x)
Supplement: Supplementary file 1 — Supplementary material [file 41420_2022_1188_MOESM1_ESM.docx]

**Supplementary Material**

**Figure S1. Ischemia-reperfusion injury upregulates the expression of LIGHT and its receptors in mouse kidney sections.** LIGHT^+/+^ mice were treated with 32 min of renal ischemia (clapping bilateral renal pedicles) followed by reperfusion for 24 or 48 h. Mice that received the same surgery without renal pedicle clamping were used as sham controls. Representative images of IHC staining for LIGHT, HVEM, and LTβR are shown. Scale bar, 50 μm.


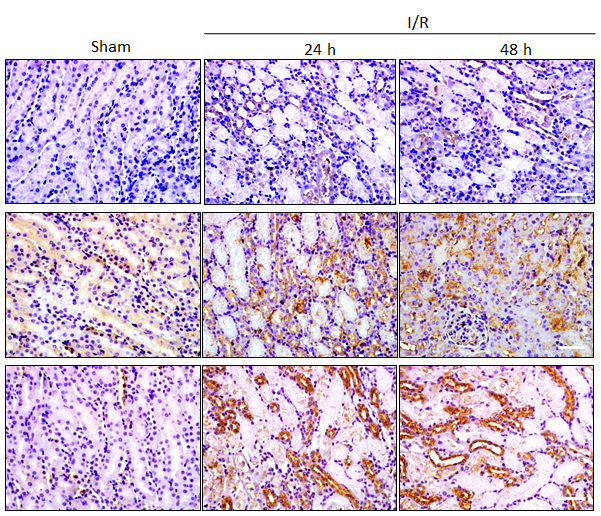


**Figure S2. Upregulated LIGHT and its receptor expression in biopsy samples with acute tubular injury.** Human kidney biopsy samples were obtained from patients after histological confirmation of acute tubular injury. Normal control renal tissues were collected from patients diagnosed with renal cell carcinoma who received radical nephrectomy surgery, and the normal peri-tumoral tissue was used. Representative images of IHC staining for LIGHT, HVEM, and LTβR. Scale bar, 50 μm.


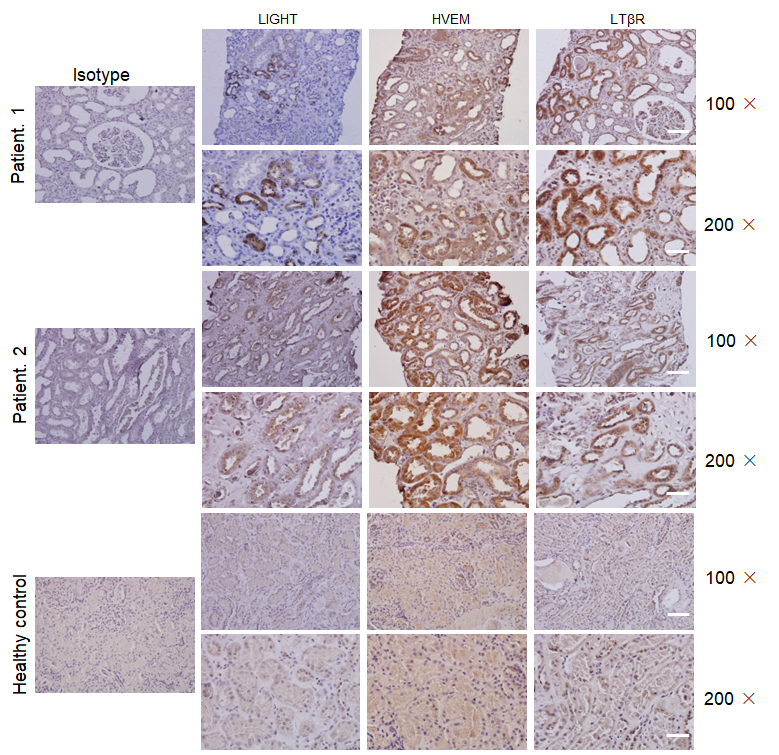


**Figure S3. LIGHT deficiency increases Bcl2 expression upon I/R injury.** Wild-type (LIGHT^+/+^) and LIGHT KO (LIGHT ^-/-^) mice were subjected to renal ischemia followed by reperfusion for 48 h. Renal samples were collected and conducted Western blotting for expression of apoptosis-related proteins (Bcl2 and Bax). Down panel: densito-metric measurements. Values are presented as mean ± SEM and data were obtained from at least two independent experiments. ns, no significance, *, p < 0.05.


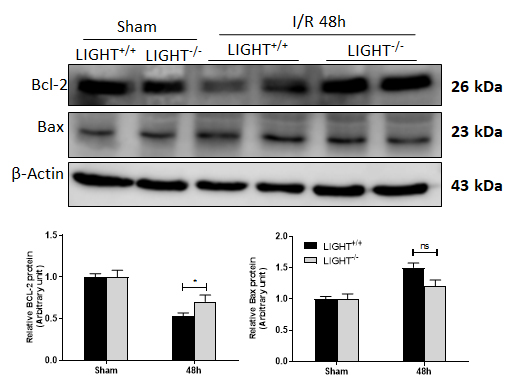


**Figure S4. LIGHT loss increases mitophagy.** LIGHT^+/+^ and LIGHT ^-/-^ mice were subjected to renal ischemia followed by reperfusion for 24 h. Renal samples were collected and conducted Western blotting for expression of mitophagy-related proteins (Ulk1 and TOMM20). Down panel: densito-metric measurements of the mitophagy proteins. Values are presented as mean ± SEM and data were obtained from at least two independent experiments. **, p < 0.01.


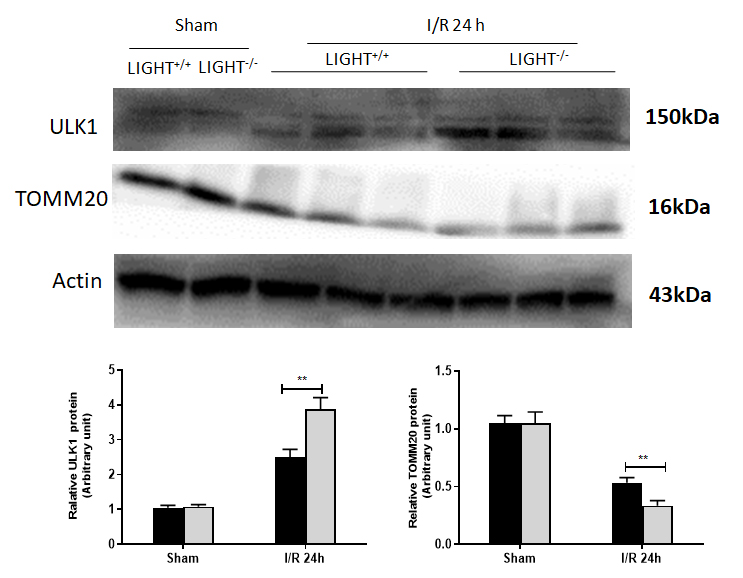


**Figure S5. Exogenous LIGHT promotes HK2 apoptosis.** HK2 cells were treated with a normal culture medium or recombinant human LIGHT and exposed to H/R conditions. Representative flow plots and graphs of apoptosis in HK2 cells are shown. *, p < 0.05; ***, p < 0.001.


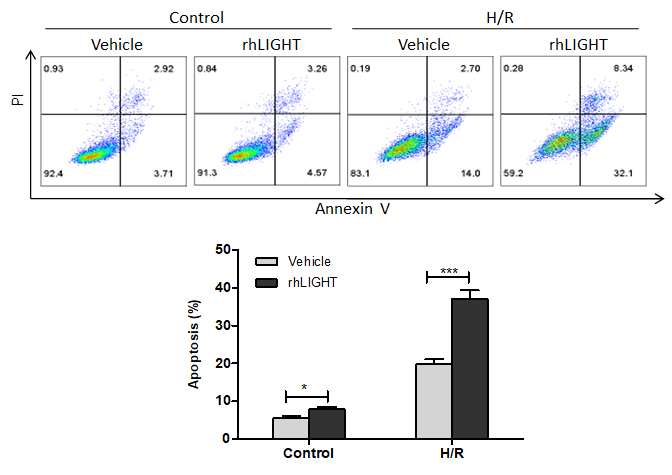


**Figure S6.** **Exogenous LIGHT increases mitochondria debris in HK2 cells.** HK2 cells were treated with a normal culture medium or recombinant human LIGHT and exposed to H/R conditions. (**A**) Representative micrographs of immunofluorescence staining for MFF (red) and COX Ⅳ (green). Quantification of colocalization of MFF and COX Ⅳ is shown in the underlying panel. (**B**) Representative micrographs of immunofluorescence staining for Mfn1 (red) and COX Ⅳ (green). Quantification of colocalization of Mfn1 and COX Ⅳ is shown in the underlying panel. Scale bar, 50 μm. *, p < 0.05; **, p < 0.01.


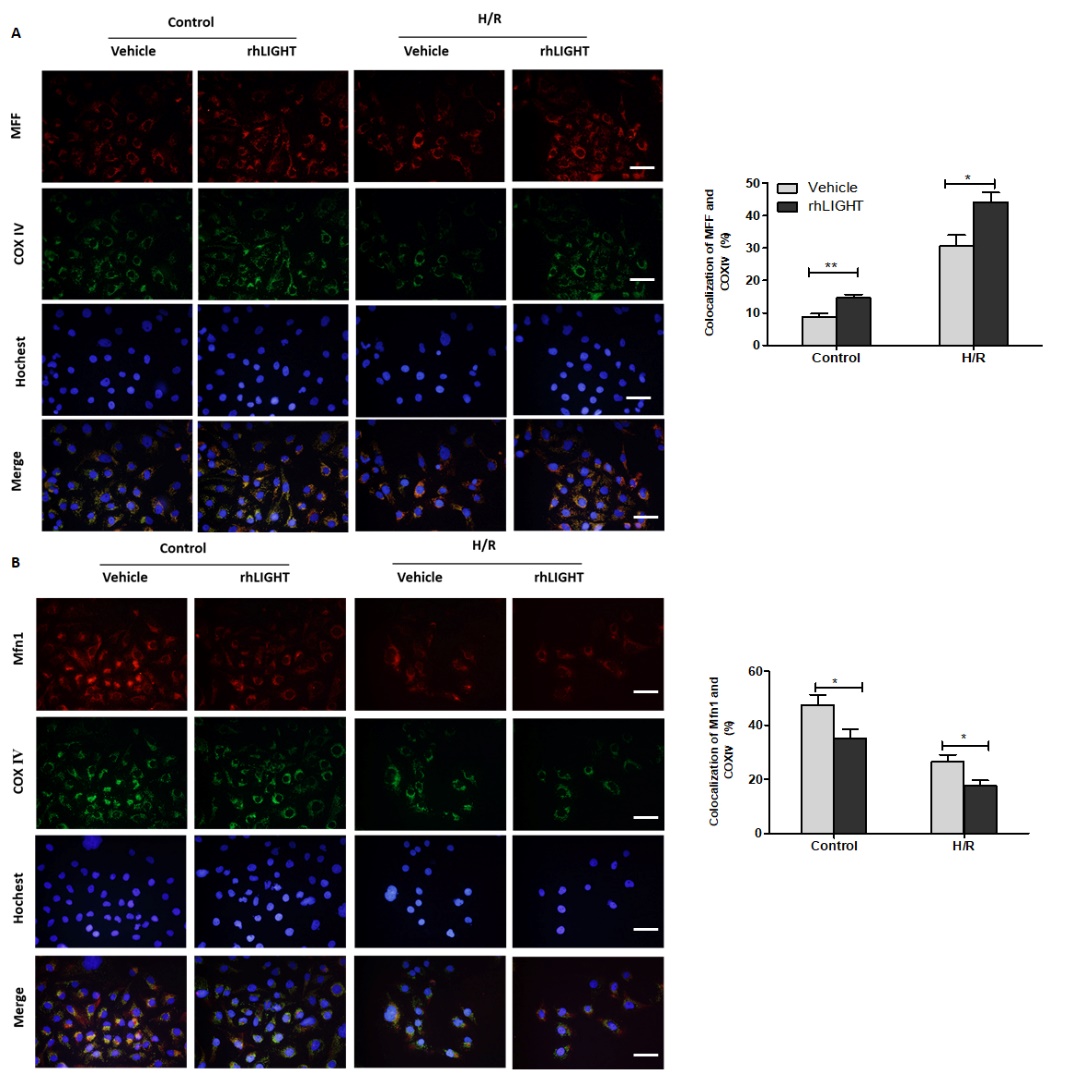


**Figure S7. Recombinant LIGHT attenuates H/R-induced mitophagy in HK2 cells.** HK2 cells were treated with a normal culture medium or recombinant human LIGHT and exposed to H/R conditions. (A) Mitophagy in each group was assessed by IF staining with LC3B (green) and Mito-Tracker (red). Representative micrographs of co-immunofluorescence mitophagy are shown. (B) Immunoblotting assay for mitophagy-related proteins, including Ulk1, Parkin, BNIP3 and TOMM20. Densito-metric analysis is shown in the underlying panel. Values are presented as mean ± SEM. Data are representative of at least three independent experiments with similar results. Scale bar, 50 μm. ns, no significance, *, p < 0.05; **, p < 0.01.


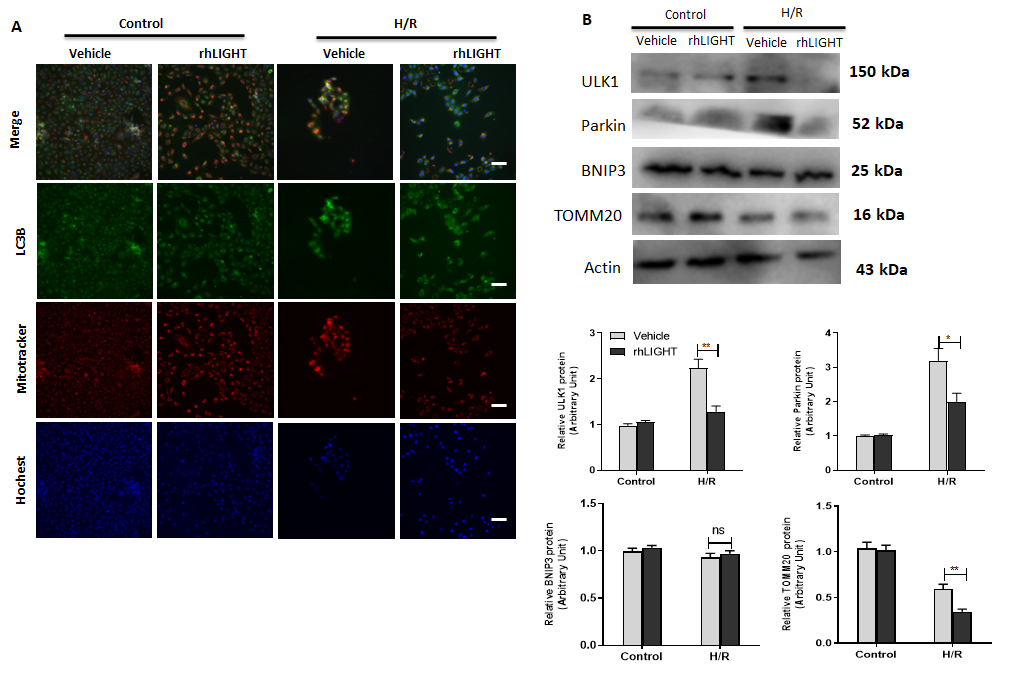


**Table 1. mRNA sequences**

| Gene | Primer sequence |
| --- | --- |
| LIGHT Forward | 5-TGGCTCCTGTAAGATGTGCTG-3 |
| LIGHT Reverse | 5-GTTTCTCCTGAGACTGCATCAA-3 |
| LTβR Forward | 5-TGCATACCGCAAAGACAAACTC -3 |
| LTβR Reverse | 5- TGGTGCCCCCTTATCGCATA- 3 |
| GAPDH Forward | 5- ACCACAGTCCATGCCATCAC-3 |
| GAPDH Reverse | 5- TCCACCACCCTGTTGCTGTA-3 |
| HVEM Forward | 5 - ACTCGTCTCCCACAAGGAACT-3 |
| HVEM Reverse | 5 - CAGGCCCCTACAGACAACAC-3 |
| IL-6 Forward | 5- GCCCTTCAGGAACAGCTATGA-3 |
| IL-6 Reverse | 5- TGTCAACAACATCAGTCCCAAGA-3 |
| KIM-1 Forward | 5-TTCTCTGTACCATGACACTCTGC -3 |
| KIM-1 Reverse | 5-ACAAGCAGAAGATGGGCATTG- 3 |
| MCP-1 Forward | 5-TTAAAAACCTGGATCGGAACCAA -3 |
| MCP-1 Reverse | 5-GCATTAGCTTCAGATTTACGGGT - 3 |
| TNF-α Forward | 5 -TCTTCTCATTCCTGCTTGTGG -3 |
| TNF-α Reverse | 5 -GGTCTGGGCCATAGAACTGA -3 |
| ND-1 Forward | 5- CTAATCGCCATAGCCTTCCTAA-3 |
| ND-1 Reverse | 5- GTTGTTAAAGGGCGTATTGGTT-3 |
| LPL Forward | 5- CCTGATGACGCTGATTTTGTAG-3 |
| LPL Reverse | 5- CAATGAAGAGATGAATGGAGCG-3 |
