# Supplementary material for: LIGHT deficiency attenuates acute kidney disease development in an in vivo experimental renal ischemia and reperfusion injury model
Source: Cell Death Discov. 2022 Sep 26;8:399. doi: 10.1038/s41420-022-01188-x (PMC9512920; doi:10.1038/s41420-022-01188-x)

Figure 1A actin

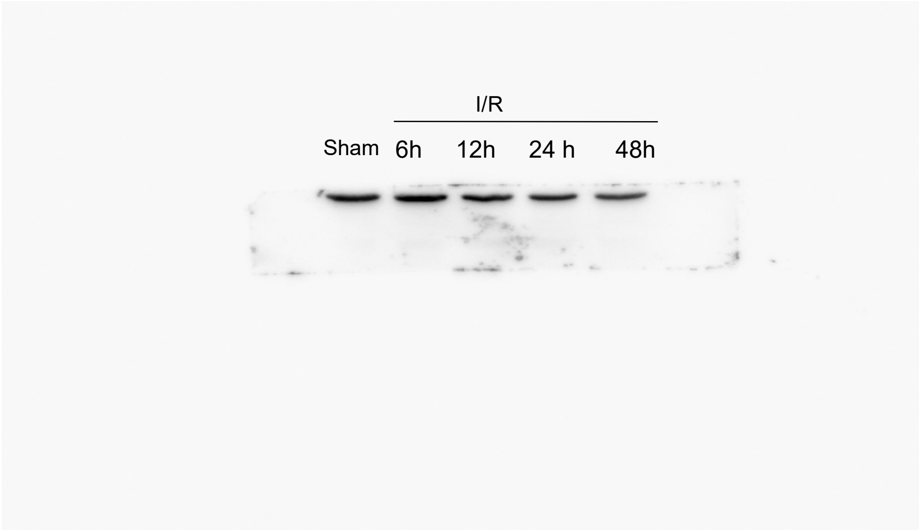

Figure 1A HVEM

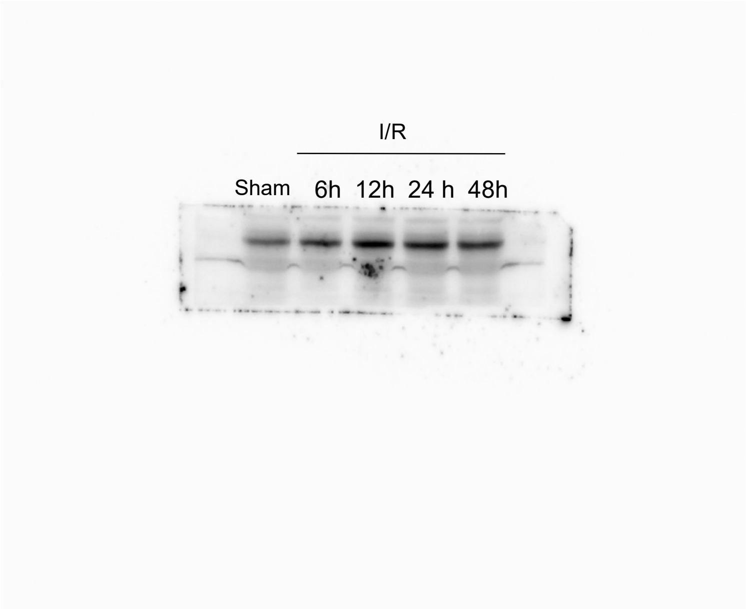

Figure 1A LTβR

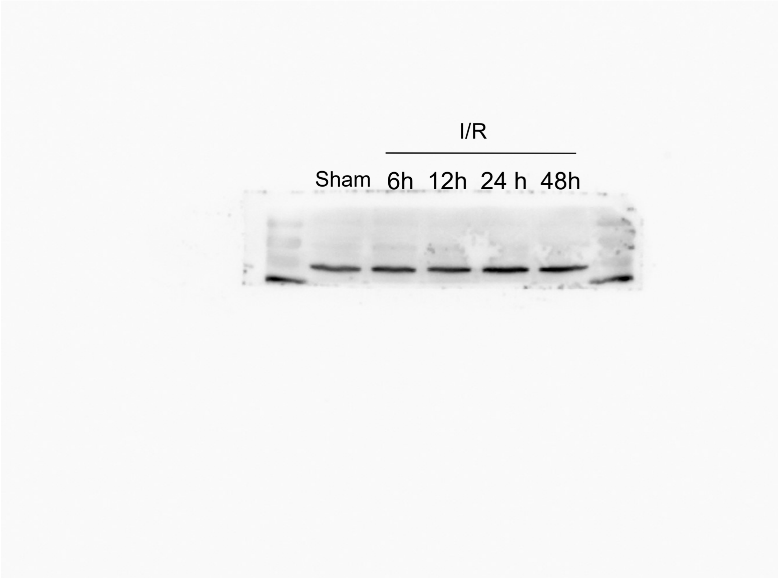

Figure 1A LIGHT

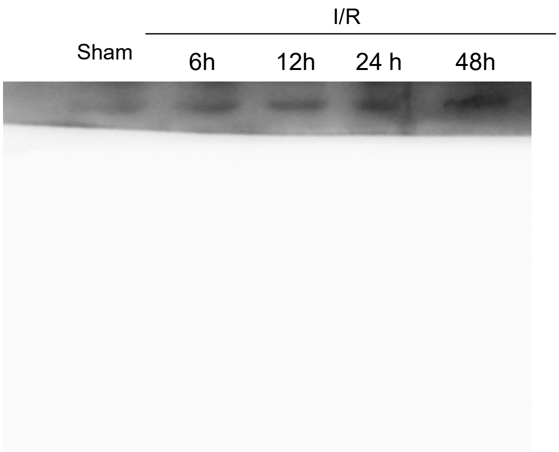

Figure 4A Bcl2

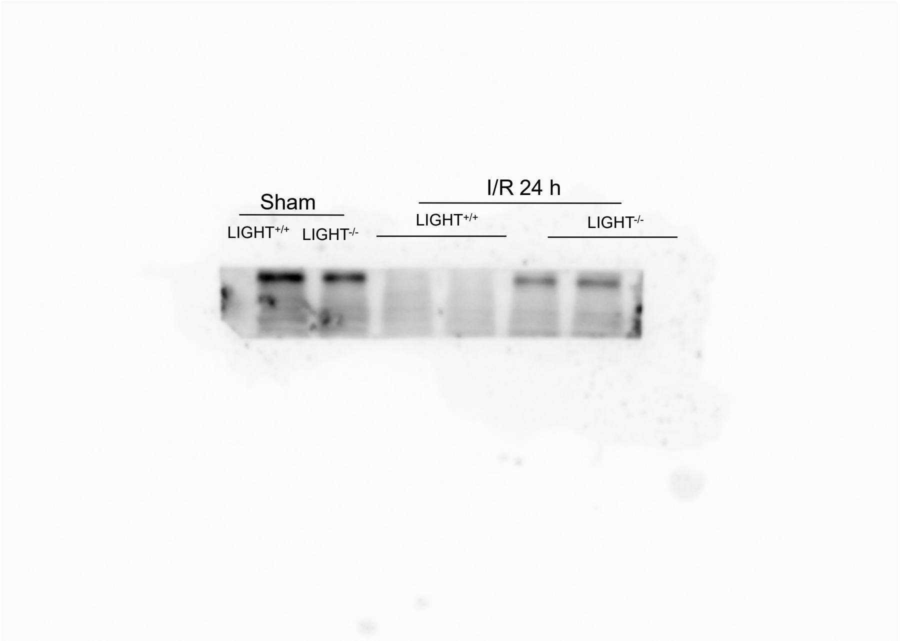

Figure 4D Bax

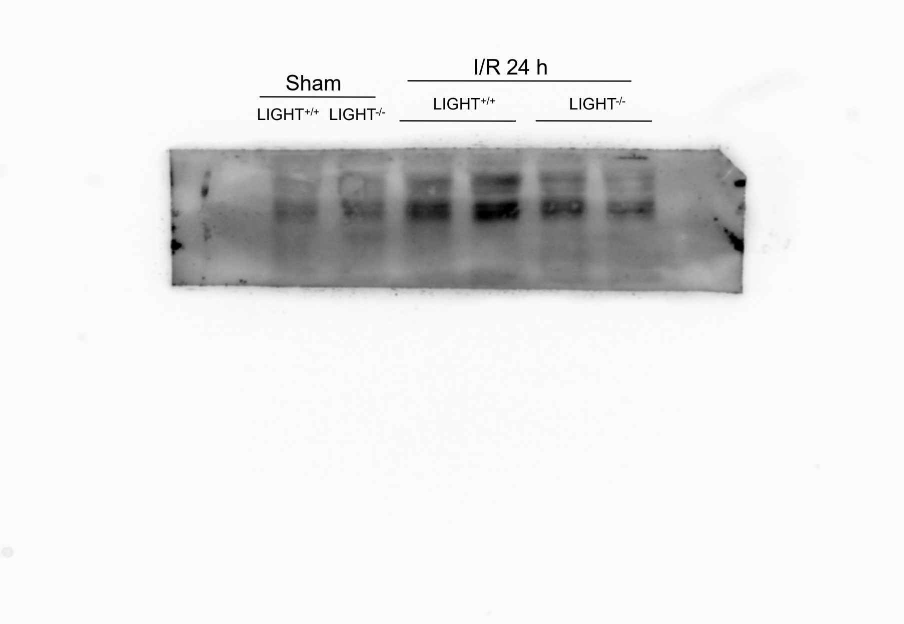

Figure 4D actin

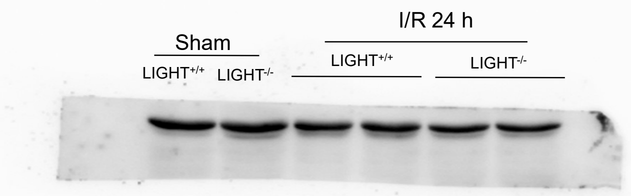

Figure 5E Drp1

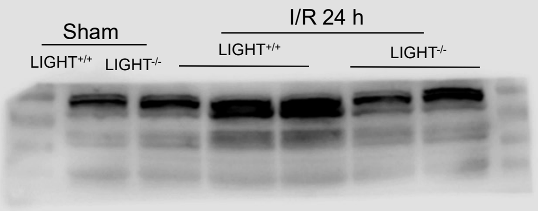

Figure 5E Mfn2

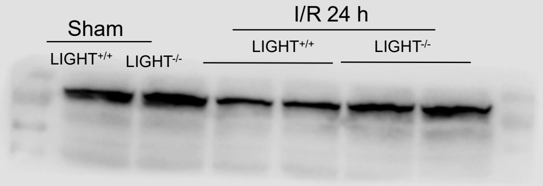

Figure 5E actin

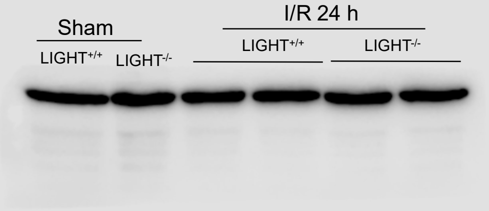

Figure 7B MFF

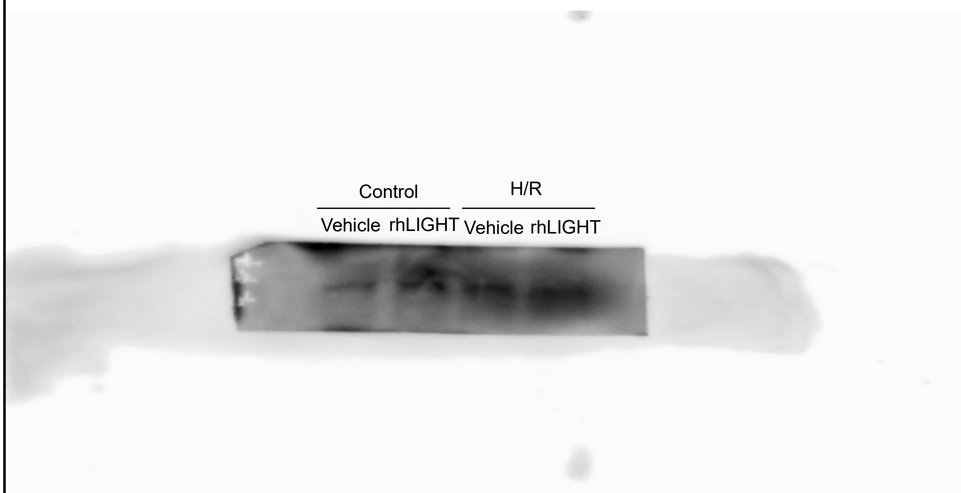

Figure 7B Mfn1

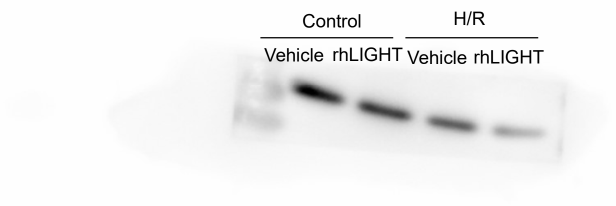

Figure 7B actin

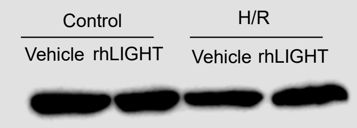

Figure 7D Drp1

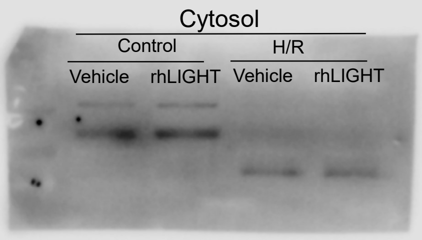

Figure 7D actin

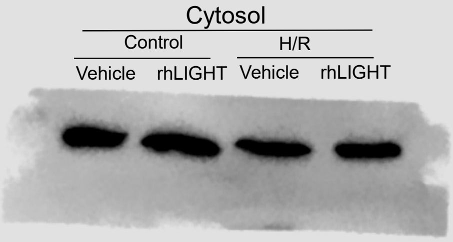

Figure 7D Drp1

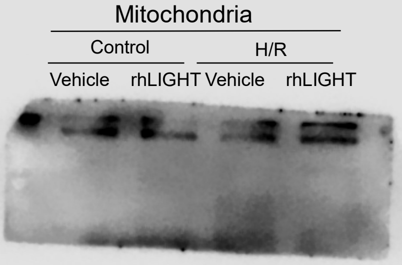

Figure 7D CoxIV

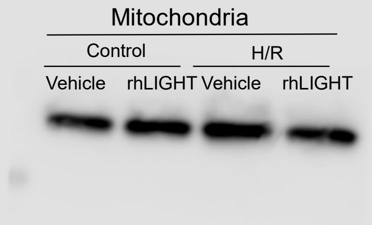

Figure 7E Drp1 p616

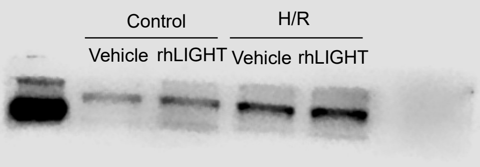

Figure 7E actin

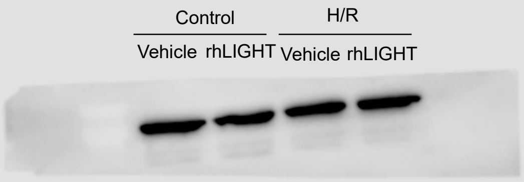

Figure 9B α-SMA

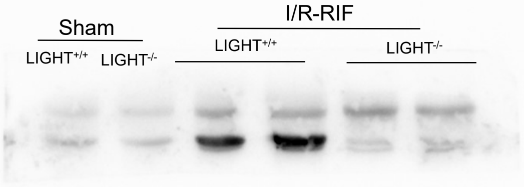

Figure 9B GAPDH

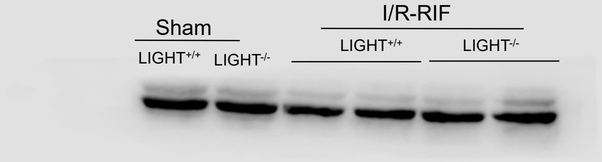

sFigure3 Bcl2

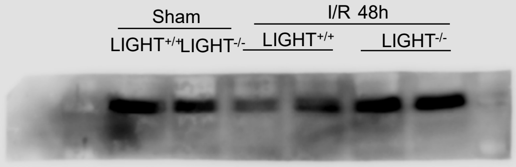

sFigure3 Bax

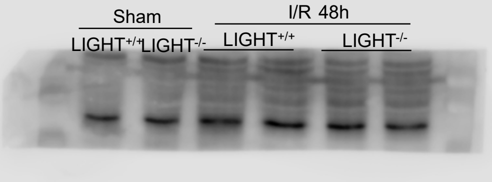

sFigure3 actin

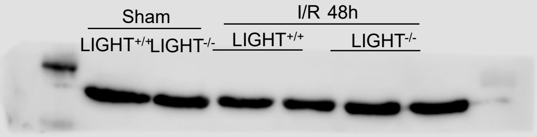

sFigure 4 ULK1

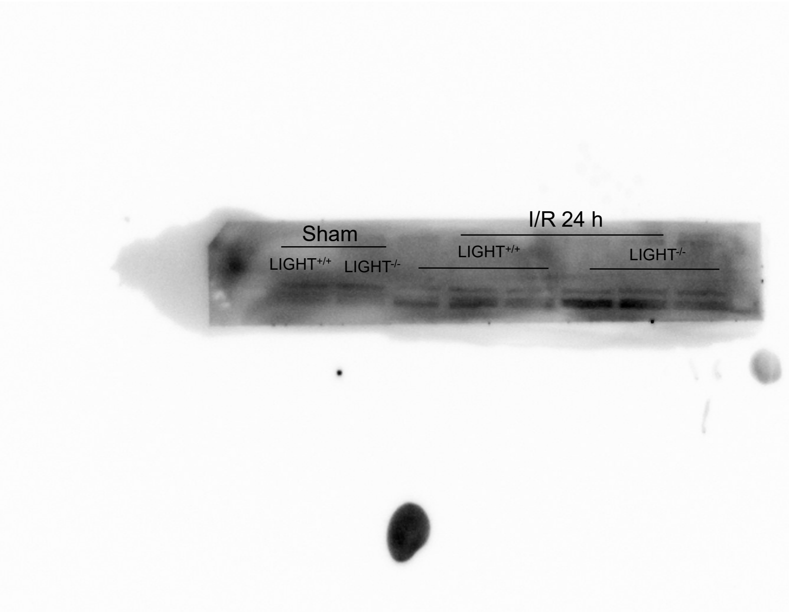

sFigure 4 Tomm20

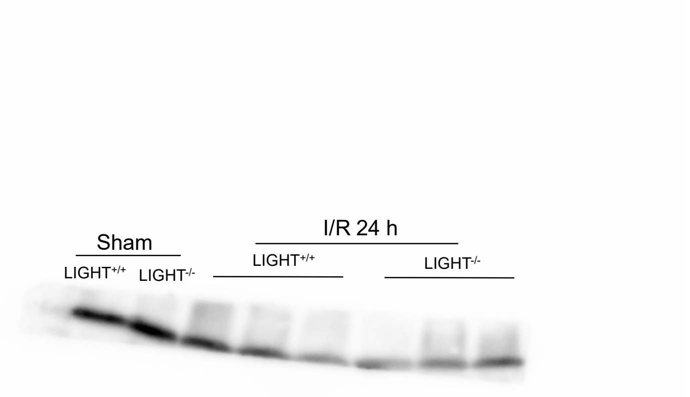

sFigure 4 actin

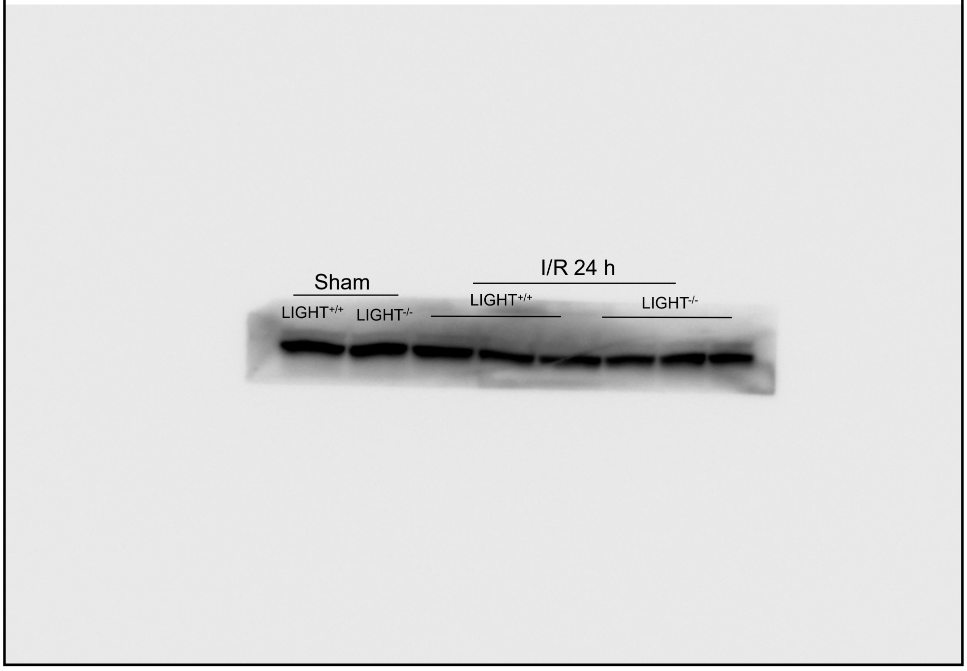

sFigure7B Ulk1

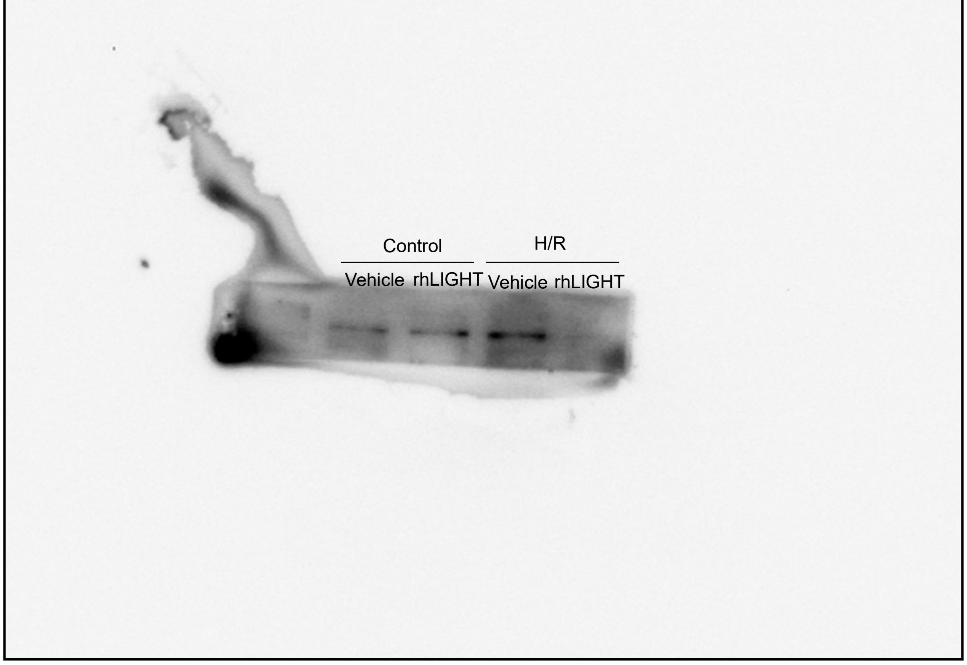

sFigure7B Parkin

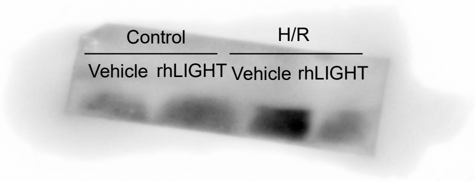

sFigure7B BNIP3

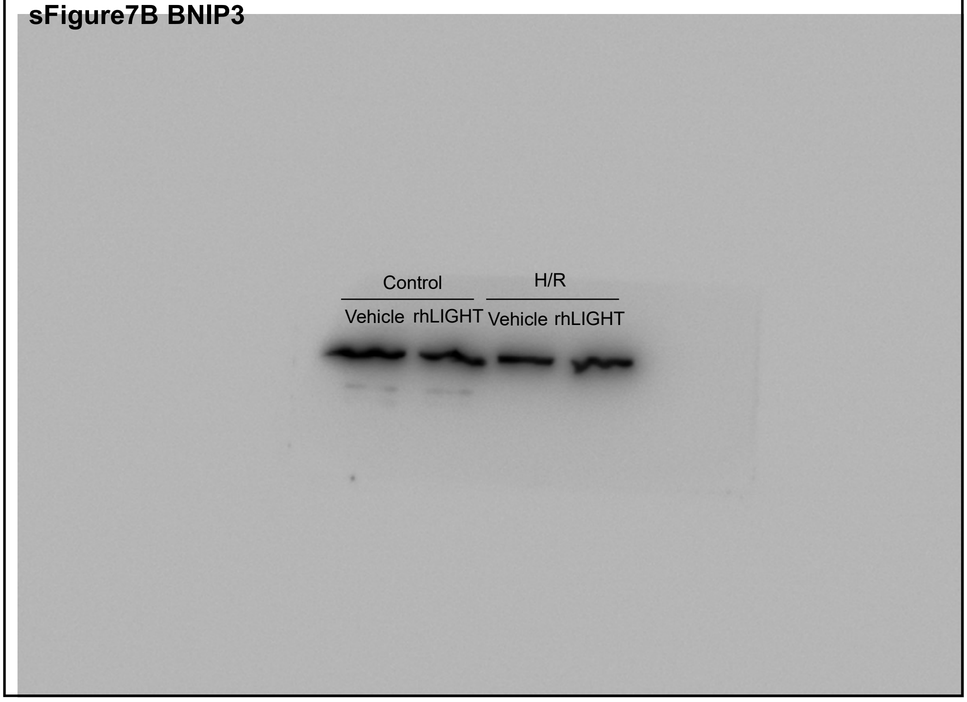

sFigure7B TOMM2O

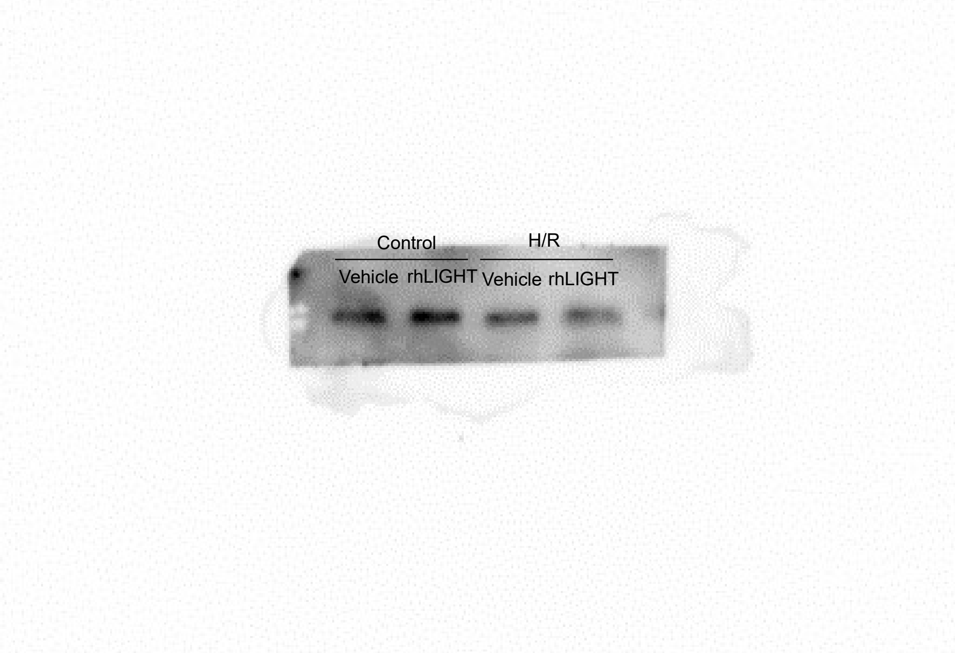

sFigure7B actin

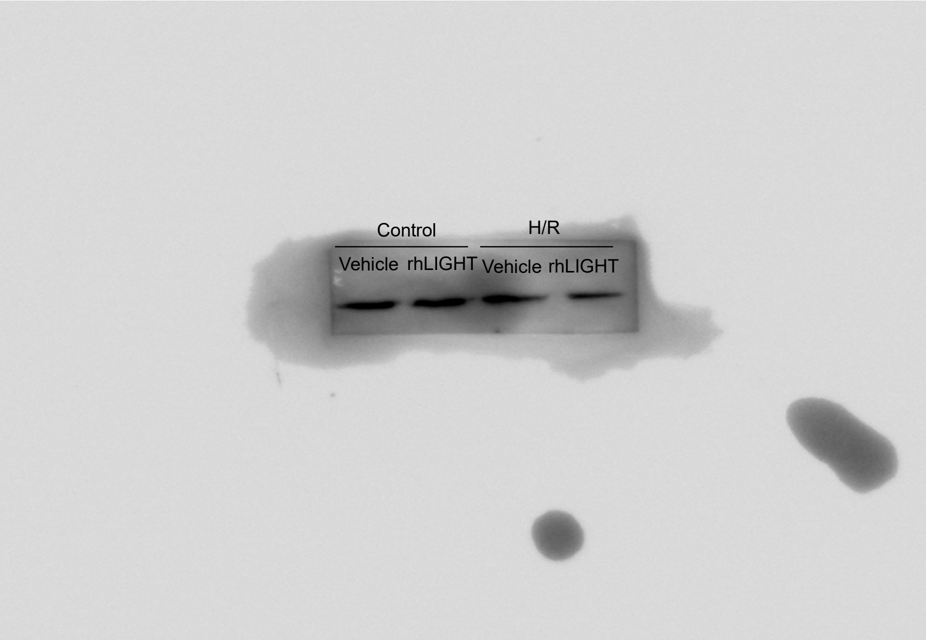

Supplement: Supplementary file 2 — Original Data File [file 41420_2022_1188_MOESM2_ESM.pdf]
